# Performance of Gut Microbiome as an Independent Diagnostic Tool for 20 Diseases: Cross-Cohort Validation of Machine-Learning Classifiers

**DOI:** 10.1080/19490976.2023.2205386

**Published:** 2023-05-04

**Authors:** Min Li, Jinxin Liu, Jiaying Zhu, Huarui Wang, Chuqing Sun, Na L. Gao, Xing-Ming Zhao, Wei-Hua Chen

**Affiliations:** a Key Laboratory of Molecular Biophysics of the Ministry of Education, Hubei Key Laboratory of Bioinformatics and Molecular-imaging, Center for Artificial Intelligence Biology, Department of Bioinformatics and Systems Biology, College of Life Science and Technology, Huazhong University of Science and Technology, Wuhan, China; b Institute of Science and Technology for Brain-Inspired Intelligence, Fudan University, Shanghai, China; c Department of Neurology, Zhongshan Hospital, Fudan University, Shanghai, China; d State Key Laboratory of Medical Neurobiology, Institutes of Brain Science, Fudan University, Shanghai, China; e MOE Key Laboratory of Computational Neuroscience and Brain-Inspired Intelligence, and MOE Frontiers Center for Brain Science, Fudan University, Shanghai, China; f International Human Phenome Institutes (Shanghai), Shanghai, China; g College of Life Science, Henan Normal University, Xinxiang, China; h Institution of Medical Artificial Intelligence, Binzhou Medical University, Yantai, China

**Keywords:** Gut metagenome, machine learning, patient stratification, cross-cohort validation, intestinal disease, metabolic disease, autoimmune disease, mental disease, liver disease, nervous system diseases

## Abstract

Cross-cohort validation is essential for gut-microbiome-based disease stratification but was only performed for limited diseases. Here, we systematically evaluated the cross-cohort performance of gut microbiome-based machine-learning classifiers for 20 diseases. Using single-cohort classifiers, we obtained high predictive accuracies in intra-cohort validation (~0.77 AUC), but low accuracies in cross-cohort validation, except the intestinal diseases (~0.73 AUC). We then built combined-cohort classifiers trained on samples combined from multiple cohorts to improve the validation of non-intestinal diseases, and estimated the required sample size to achieve validation accuracies of >0.7. In addition, we observed higher validation performance for classifiers using metagenomic data than 16S amplicon data in intestinal diseases. We further quantified the cross-cohort marker consistency using a Marker Similarity Index and observed similar trends. Together, our results supported the gut microbiome as an independent diagnostic tool for intestinal diseases and revealed strategies to improve cross-cohort performance based on identified determinants of consistent cross-cohort gut microbiome alterations.

## Introduction

In recent years, the human gut microbiome is emerging as a relevant factor in human diseases. For example, dysbiosis of the gut microbiome, i.e., the significant deviation of the gut microbiota compositions in disease subjects as compared with healthy controls, has been linked to multiple human diseases such as the intestinal^1–6^, autoimmune^7–9^, metabolic^10–13^, neurological and mental diseases^14–19^, and others^20–24^. To explore such associations, a case–control study is often carried out that involves: 1) recruiting volunteers of a disease of interest and matching health or non-disease controls (a cohort), 2) collection of fecal samples, followed by next-generation sequencing of either the 16S rRNA genes (16S) or the whole metagenome (mNGS), 3) bioinformatics analysis to determine the microbial compositions of samples, i.e., microbial taxa and their relative abundances, and 4) identification of differentially abundant microbial taxa between the case and control groups known as disease biomarkers^25–27^.

In addition, the modulatory or causal roles of gut dysbiosis have been experimentally validated in many diseases. For example, disease symptoms and/or characteristics could be reproduced in model animals by transplanting feces from patients/disorder mice of Autism Spectrum Disorder (ASD)^28^, Alzheimer’s Disease (AD)^29,30^, Obesity^31^, and Diabetes^32^. On the other hand, restoring the gut microbiota by transplanting feces from healthy donors to human recipients alleviated symptoms in diseases such as Clostridium Difficile Infection (CDI)^33^, Inflammatory Bowel Disease (IBD)^34^, and ASD^35^.

Alterations in the human gut microbiome thus have been increasingly used as biomarkers for noninvasive disease prescreening and diagnosis, and targets for disease treatment and intervention. For disease diagnostic purposes, machine learning (ML) classifiers are also often trained on either the microbial compositions alone or in combination with clinically relevant features to distinguish patients from controls^36–38^. These ML models are often validated on holdout samples of the same cohort to evaluate predictive performance as the area under the receiver operating characteristic curve (AUCs) (i.e., intra-cohort validation), or in rare cases, on independent cohorts for cross-cohort validation^39^. Among the ML algorithms, Random Forest and Least Absolute Shrinkage and Selection Operator (Lasso) logistic regression-based approaches are the most popular ones because of their advantages including high performance on smaller sample sizes (e.g., less than 50), complex and heterogenous data (e.g., high-dimensional composition data)^36^, explicit ranking on feature importance, and low overfitting risks by feature selection. So far, gut microbiome-based diagnostic classifiers have been available for CRC^1,39^, IBD^40^, Liver Cirrhosis (LC)^41,42^, Pancreatic Ductal Adenocarcinoma (PDAC)^43,44^, ASD^45^, AD^15,46,47,^ and many others^48–50^.

For disease intervention and treatment, using fecal microbiota transplantation (FMT) from healthy donors^33^, and targeting the depleted/enriched microbial biomarkers in patients have been used. For example, supplementing mice with the *Lactobacillus murinus* strain could decrease high salt-sensitive hypertension, and in solid tumor models, the application of a mix of commensal gut *Clostridiales* strains in mice could enhance anti-cancer immune responses^51,52^. Conversely, *Duan* et al. used bacteriophage targeting of a patient-enriched bacterium to decrease cytolysin in the liver and abolish ethanol-induced liver disease in mice transplanted with microbiota from alcoholic liver disease patients^53,54^. In addition, Type 2 Diabetes Mellitus (T2D)-deficient species could be selectively promoted by designed diets (i.e., dietary fibers) for treatment^55^.

However, controversies exist with the reproducibility of gut dysbiosis in different cohorts, because the gut microbiome is known to be easily and significantly affected by external factors including diet^56^, drugs^57,58^, regional differences^59^, sample preprocessing processes^60^, and data analysis methods^61,62^. These confounding factors often vary among cohorts, and sometimes dominate the gut microbiome alterations. For example, *Chloe* et al. revealed that the gut microbiome alterations in ASD children compared with paired siblings in an Australian cohort were mostly due to dietary preferences^63^. Moreover, common prescription drugs such as metformin for T2D^12,64^, proton pump inhibitors (PPIs) for gastrointestinal (GI) disorders^65^ and LC^66^, and statin for Obesity^67^ and Cardiovascular Disease (CVD)^58^, could dominate the gut microbiome alterations over the corresponding diseases, either alone^12,66^ or in combination^58^. In addition, disease biomarkers could also differ significantly across cohorts of the same diseases^68^. These controversies would greatly impede the real-life applications of the research results for disease diagnosis and targeted intervention.

There is thus an urgent need to test and validate the cross-cohort reproducibility of the gut microbiome as diagnostic prescreening tools, and the cross-cohort consistencies in disease biomarkers. Researchers have recently started such investigations in individual diseases, and revealed higher cross-cohort prediction validation results in CRC^39^ and LC^42^, in contrast to lower performances in Adenoma^69^, T2D^69,^ and psychiatric disorders^70^. However, a systematic evaluation of cross-cohort reproducibility of gut microbiome alternations in all available datasets is yet to be performed^71–75^; in addition, the influential factors (i.e., determinants) of the reproducibility are yet to be explored^76^.

In this study, we conducted a comprehensive meta-analysis for 20 diseases, using 83 case–control cohorts with a total of 9,708 samples; these diseases spanned five major disease categories, with each disease having two or more cohorts. We performed intra-cohort and combined-cohort modeling and predictive validations using state-of-the-art tools, accessed factors affecting the prediction accuracies, and recommended strategies to improve cross-cohort validation performance in order to support the gut-microbiota-derived classifiers as disease prescreening tools.

## Results

### Selection of gut microbiome cohorts and modeling strategies

**Figure 1.**
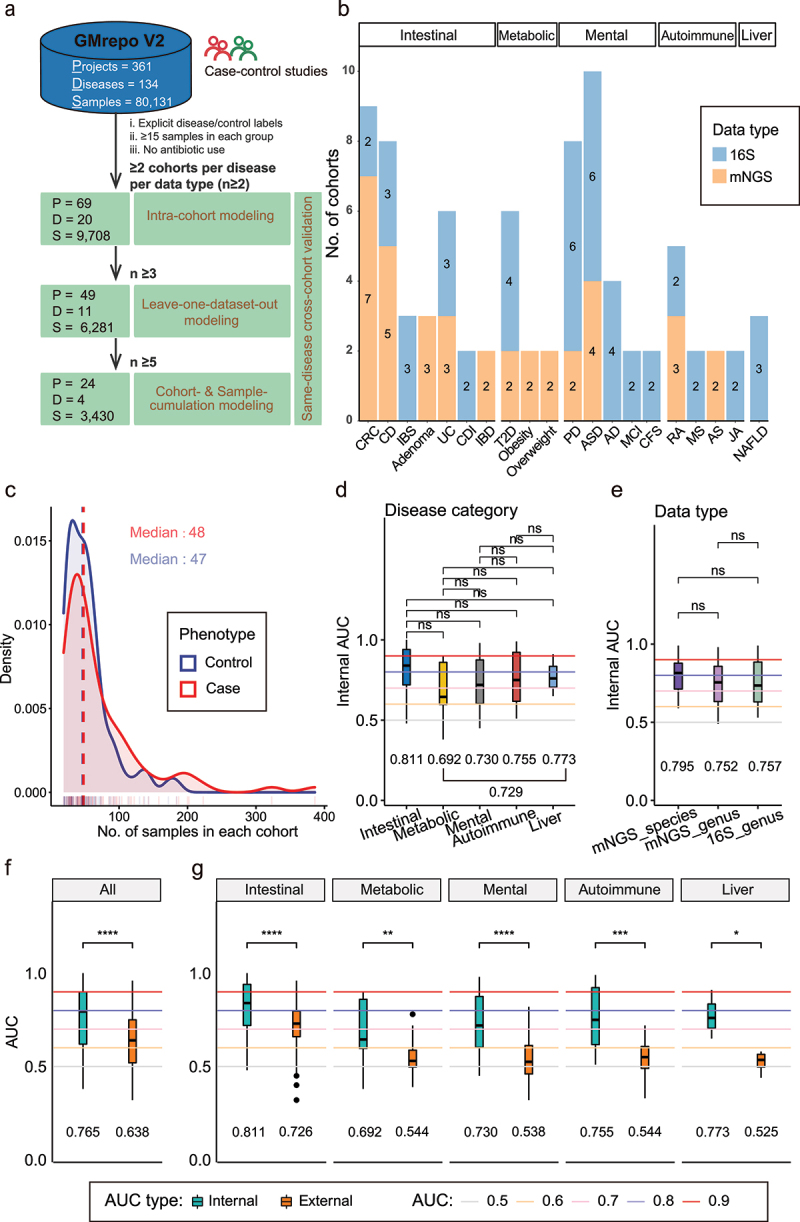
Study design, information of datasets and intra-cohort validation result. (a) Overview of analysis workflow. 361 human gut microbiome case-control (controls only include health phenotype) studies about 134 diseases from a public database were preserved, of which 69 projects about 20 diseases were ultimately selected. Then different modeling methods and cross-cohort (external) validation on the same disease and data type were performed, which are influenced by the cohort size (n) of same disease. First, all diseases with *n* ≥ 2 were enrolled by intra-cohort modeling (i.e., building single-cohort classifiers). Second, only diseases with *n* ≥ 3 were performed leave-one-dataset out (LODO) analysis (one of combined-cohort modeling). Thirdly, only diseases with *n* ≥ 5 were enrolled by cohort-cumulation modeling (CCM) and sample-cumulation modeling (SCM) analyses (two of combined-cohort modeling, Methods). (b) Disease information about filtered 83 cohorts. There are five broad categories of diseases, where Mental diseases represent Mental and Nervous system diseases. Colors represent different data types. The numbers on the graph represent cohort size for each disease on each data type. (c) Density plot of the No. of samples in each cohort. The median sample sizes of case and control are 48 and 47, marked by the red and blue lines, respectively. (d) Comparation of internal validation AUCs with intra-cohort modeling between different disease categories. Multiple adjusted two sides Wilcoxon rank sum test was used for pairwise group comparisons. (e) Comparation of internal validation AUCs with intra-cohort modeling between three different data types (Only diseases with both 16S and mNGS sequencing types were included). (f) Comparation of AUCs with intra-cohort modeling between internal and external validations in overall. Two sides Wilcoxon rank sum test was used for comparisons. (g) Comparation of AUCs with intra-cohort modeling between internal and external validations in Intestinal, Metabolic, Mental, Autoimmune and Liver five disease categories. Two sides Wilcoxon rank sum test was used for pairwise group comparisons. The colored horizontal lines represent different AUC levels. The numbers marked in the bottom of d, e, f and g represent the mean of the corresponding AUCs. *p < 0.05, **p < 0.01, ***p < 0.001, ****p < 0.0001.

To select gut microbiome data for cross-cohort validation, we screened a total of 361 studies in the GMrepo v2 database that were systematically collected, manually curated, and consistently analyzed^68^ (Figure 1a). Our inclusion criteria included: 1) case–control studies with clearly defined disease information, 2) with at least 15 valid samples in each of the case and control groups (Methods), 3) no recent use of antibiotics or probiotic supplements. We divided the qualified cohorts into two groups accordingly to their sequencing methodology, namely 16S for 16S ribosomal RNA gene amplicon sequencing, and mNGS for whole-metagenomic shotgun sequencing (here NGS stands for next-generation sequencing). We further required that a disease should have at least two cohorts in the 16S or mNGS groups. In the end, we obtained 83 cohorts from 69 studies (some studies contained two and more diseases) that contained in total 5,984 cases and 3,724 non-disease controls. Most of the cohorts were sequenced using the Illumina platforms (Table S1). These cohorts included 20 diseases; among which, eight were unique to the 16S group, including Irritable Bowel Syndrome (IBS), CDI, AD, Mild Cognitive Impairment (MCI), Chronic Fatigue Syndrome (CFS), Multiple Sclerosis (MS), Juvenile Arthritis (JA), Non-alcoholic Fatty Liver Disease (NAFLD); five were unique to the mNGS group, including IBD, Obesity, Overweight and Ankylosing Spondylitis (AS) and Adenoma; and seven had more than two cohorts in both groups, including Crohn Disease (CD), Colorectal Cancer (CRC), Ulcerative Colitis (UC), T2D, Parkinson’s Disease (PD), ASD and Rheumatoid Arthritis (RA) (Table S1, Figure 1b). We divided these 20 diseases into five categories, including seven Intestinal, three Metabolic, four Autoimmune, five Mental/nervous system diseases (Mental for short), and one Liver disease (Figure 1b, Table S1; Methods), according to the NCBI MeSH (Medical Subject Headings) database and Human Disease Ontology (DO) database^77^. These cohorts could contain up to 323 cases and 184 controls; however, most studies have been conducted on limited numbers of samples, with median sizes of 48 and 47 for the cases and controls, respectively (Figure 1c).

For mNGS data, we analyzed their taxonomic relative abundances at both species and genus levels. However, due to the limited taxonomic resolution, we only analyzed the genus-level profiles of the 16S sequencing data. To control for intra-cohort confounding factors, we tested different distributions of the age, gender, body mass indexes (BMI), disease stage and geography between the case and control groups for each cohort, and adjusted the microbial compositions of the gut microbiome data for those with p-values<0.05 using *removeBatchEffect* function implemented in the ‘limma’ R package (Methods). We further removed cross-cohort batch effects using the *adjust_batch* function implemented in the ‘MMUPHin’ R package by using the project-id as the controlling factor.

To select the best ML algorithm, we evaluated four algorithms that were popular in gut microbiota studies, including Elastic Network (Enet)^78^, Lasso^79^, Random Forest (RF)^80^ and Ridge Regression (Ridge)^81^ (Methods). We tested them on all datasets, among which CRC and IBD that have been extensively investigated in both individual cohorts and meta-analyses^6,39,40,82^. We first performed intra-cohort modeling (building single-cohort classifiers) and evaluation using five-fold three times cross-validations (5-fold 3 times; Methods). We then performed cross-cohort validation with applying the single-cohort classifiers to other cohorts of the same diseases, and measured the predictive performances as AUCs (area under the receiver operating characteristic curve). We obtained similar results in terms of internal and external validation AUCs with intra-cohort modeling for the four ML algorithms on selected diseases (Fig. S1A; Methods). In the end, we chose the Lasso algorithm for all subsequent analyses.

We also examined whether feature selection could improve the intra-cohort and/or cross-cohort validation of single-cohort classifier performances. To avoid over-fitting issues caused by label leakage, we adopted a nested feature selection strategy as recommended by *Wirbel* et al.^83^ (Methods). When applying this strategy to five selected diseases, including CRC, CD, ASD, PD and AD, we found both internal and external AUCs were increased with the increasing top feature size in general (Fig. S1B), and selecting top features did not significantly improve the internal and external AUCs (Fig. S1C). Thus, we used all gut microbial features for ML modeling and validation in the subsequent analyses. In addition, we also found that logarithmically transforming the relative abundance data could significantly improve the external AUCs (Fig. S1D, *p* = 1.8e-06, paired Wilcoxon rank sum test) and marginally the internal AUCs (*p* = 0.074; Methods). Thus, we used the logarithmically transformed data in all subsequent analyses.

In addition to intra-cohort modeling, we also performed three combined-cohort modeling for diseases with required numbers of available cohorts (Figure 1a; Methods). First, for diseases with more than three cohorts, we performed a leave-one-dataset out (LODO)^39^ analysis by training the model on the pooled samples from all cohorts except the one used for model testing (Methods). Second, for diseases with more than five cohorts, we performed a cohort-cumulation modeling (CCM) analysis by randomly combining increasing number of datasets for training and then testing the remaining cohorts in the same disease (Methods), and a sample-cumulation modeling (SCM) analysis by combining increasing numbers of samples randomly selected from the LODO training dataset as the training data and then testing the resulting classifiers on the remaining cohort of the same disease (Methods). These analyses helped us to determine whether including more samples from multiple cohorts could improve the predictive validation performances, and the minimal sample sizes required to achieve certain AUC levels.

### Gut microbiome-based classifiers have high intra-cohort predictive accuracies with mean AUC 0.77

To test whether taxonomic relative abundances of the gut microbes (i.e., features) could be used to distinguish the cases from controls within each cohort, we first built Lasso classifiers using all features (excluding samples with only two taxa or fewer and low-abundant taxa; Methods) and validated their performance using five-fold three times repeated intra-cohort cross-validation (internal validation, Methods). For 16S cohorts, genus-level relative abundances were used; for mNGS cohorts, two classifiers were built for each cohort using genus- and species-level relative abundances, respectively. In total, we obtained 120 classifiers for the 83 cohorts. We observed decent predictive performances averaging at 0.77 AUCs (Q1, First Quartile: 0.62; Q3, Third Quartile: 0.90; SD, Standard Deviation: 0.16) (Table S2). When grouping disease into five categories according to the NCBI MeSH database, we observed no significant differences among the five categories, namely, Intestinal, Metabolic, Mental, Autoimmune and Liver disease (Figure 1d). However, the Intestinal diseases showed the highest mean intra-cohort validation AUC of 0.811, while the Metabolic diseases showed the lowest (0.692; Figure 1d).

These intra-cohort AUCs were largely consistent with those reported in the works of literature (Fig. S1E, Table S5), except for three projects which our AUCs were significantly lower (Fig. S1E, indicated by red circles). Among them, the PRJNA686821 (ASD)^84^ and PRJNA496408^46^ (including MCI and AD disease samples) used a feature selection procedure that cause label leakage and artificially increase intra-cohort AUCs^85^. For the PRJEB13092 (CFS)^86^, the higher AUC reported by the literature was mostly due to using additional meta-data in the model training, which accounted for the top three most important features^86^ (Table S5).

In addition, we found comparable internal AUC values among data types, although the AUC values were slightly higher when mNGS data-derived species-level relative abundances were used (Figure 1e).

Together, we showed that gut microbiome-based patient-stratification classifiers could have high intra-cohort predictive performances for the 20 diseases.

### Prediction on independent cohorts leads to significantly reduced accuracy, except for intestinal diseases

**Figure 2.**
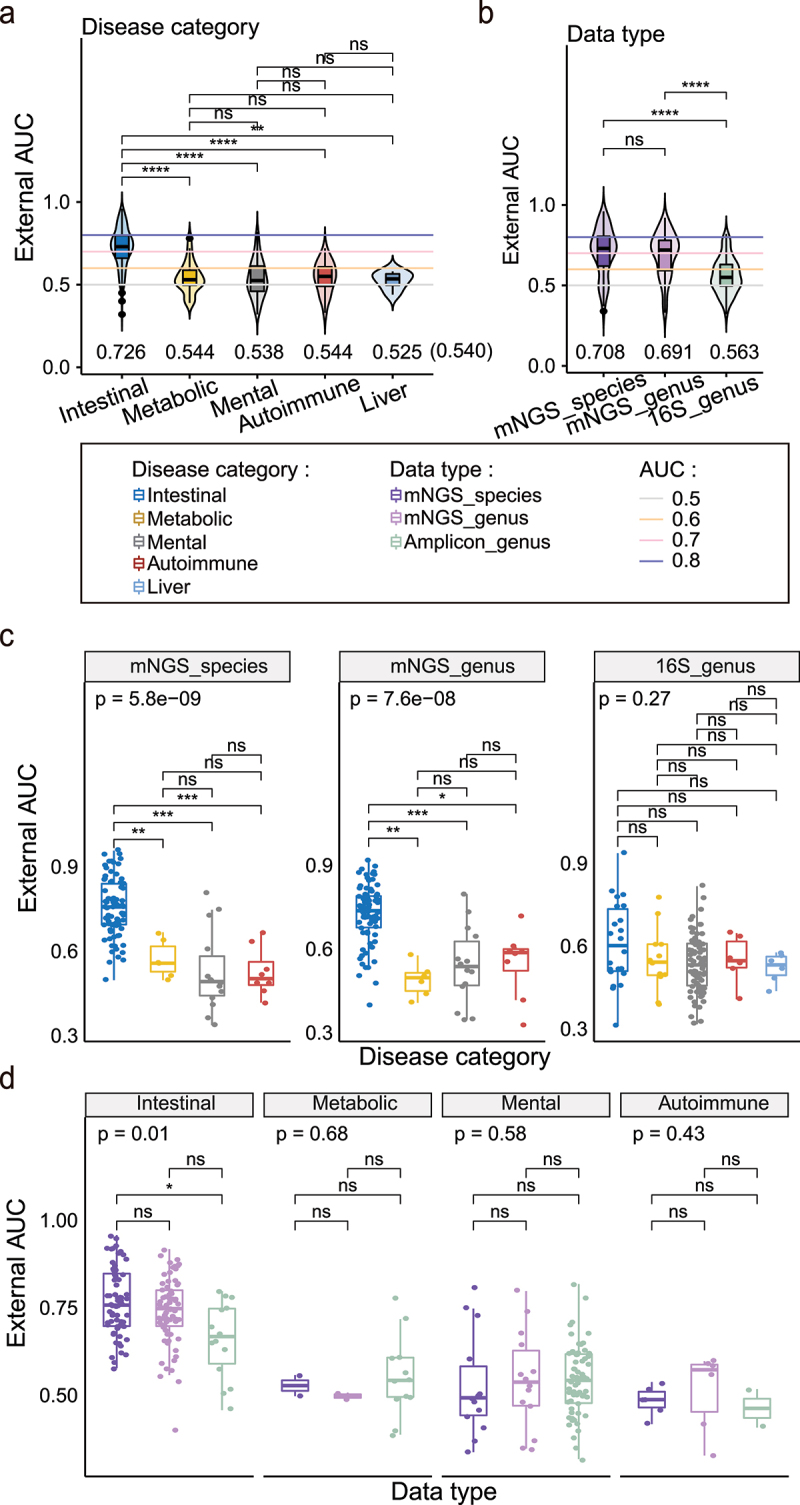
Comparation of external validation with intra-cohort modeling under different disease categories and data types. (a) Comparation of external validation AUCs with intra-cohort modeling between five different disease categories. Multiple adjusted two sides Wilcoxon rank sum test was used for pairwise group comparisons. Kruskal–Wallis test was used for multiple-group comparisons (p < 2.2e − 16). (b) Comparation of external validation AUCs with intra-cohort modeling between three different data types. Kruskal–Wallis test was used for multiple-group comparisons (p = 2.5e − 12). (c) Boxplots of external validation AUCs under different disease categories in each data type. Points represent the external validation AUCs, and colors represent the different disease categories. Kruskal–Wallis test was used for multiple-group comparisons and *p* values were shown at the top of the picture. Multiple pairwise Wilcoxon rank sum test comparisons were adjusted and *p* values were shown above the line segment. Box elements show the median and upper and lower quartiles. (d) Boxplots of external validation AUCs between different data types in each disease category. BD (Only diseases with both 16S and mNGS sequencing types were included.

We then examined the predictive performance of the single-cohort classifiers on independent cohorts of the same diseases. We obtained in total 330 external validation AUCs with intra-cohort modeling and observed significantly decreased validation performances with an average AUC of 0.64 (Q1: 0.52, Q3: 0.76, SD: 0.15) compared with the intra-cohort validations (Figure 1f, *p* = 1.7e-12, Wilcoxon rank sum test). The decreases could be found in all disease categories (Figure 1g), and there were significant differences between disease categories (Figure 2a, *p* < 2.2e-16, Kruskal Wallis test); for example, we obtained decent external validation AUCs for the Intestinal diseases with an average of 0.73 (Q1: 0.65, Q3: 0.79; SD: 0.12; Figure 2a), which was above the acceptable level of discrimination power (e.g., AUC>0.7). By contrast, the average AUCs in the other four disease categories dropped to ~ 0.54 (Q1: 0.47, Q3: 0.61; SD: 0.11; Figure 2a, Table S2), which was only slightly better than a random guess. Overall, the cross-cohort validation AUCs of single-cohort classifiers on intestinal diseases were significantly better than the other four disease categories (Figure 2a). Of note, we also calculated alternative model performance measurements for both the intra- and cross-cohort analysis, including AUC-PR (the area under the precision-recall curve) and MCC (Mathews Correlation Coefficient) (Methods), and observed consistent trends. In fact, all three measurements (i.e., the AUC, AUC-PR, and MCC) showed strong pairwise correlations (Fig. S2).

We speculated that the high external AUCs in the intestinal diseases were due to the direct interactions between the diseased sites and the gut microbiota. For example, CRC, CD, and IBD are often associated with physiological/pathological changes in the intestine^87^, which will render direct and significant impacts on the gut microbiota. So, they dominate the effects over other biological and technical factors, and facilitate cross-cohort validation. This line of reasoning predicts that intestinal diseases that are either at early disease stages (i.e., Adenoma) or often dormant (i.e., IBS) that do not significantly change the intestine and/or the gut microbiome, should have lower cross-cohort validation results. As expected, we observed high external validation performances for CD, IBD, and CRC with averaging AUCs of 0.79, 0.77, and 0.74, respectively, in contrast to much lower external AUCs of 0.59 and 0.547 for Adenoma and IBS (Fig. S3; Table S2).

Together, we showed applying single-cohort ML classifiers to independent cohorts generally led to significantly decreased predictive performance. In addition, we identified the disease category as a key determinant for reproducible gut microbiome-based disease classifiers.

### mNGS-based classifiers perform better than 16S-based ones in cross-cohort validation in intestinal diseases

During the external validation analysis of the single-cohort classifiers, we observed significantly higher performances of the mNGS-based classifiers than the 16S-based ones: as shown in Figure 2b, the AUCs of both the mNGS species- (mean: 0.71, SD: 0.15) and genus- (average: 0.69, SD: 0.14) level classifiers were higher than 16S (mean: 0.56, SD: 0.11). These results implied that data type could also be a determinant factor in cross-cohort validation.

**Figure 3.**
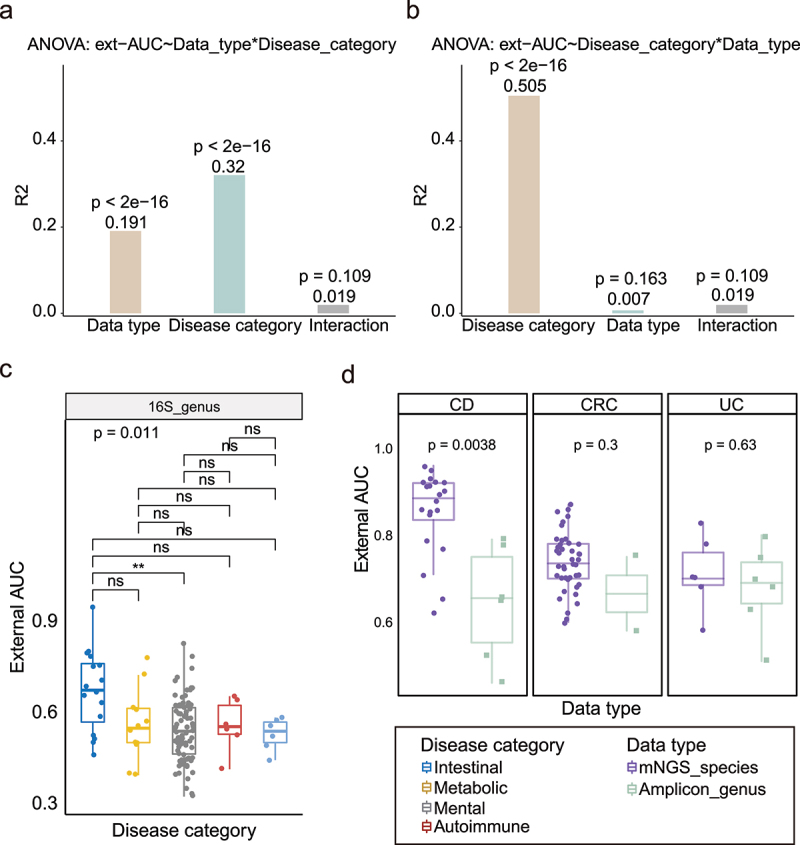
ANOVA analysis and comparation of external validation with intra-cohort modeling in detail. (a) Two-factor with interaction (data type * disease category) ANOVA of external AUCs. The R^2^ and *p* values of the factors were shown above the box. (b) Two-factor with interaction (disease category * data type) ANOVA of external AUCs. The R^2^ and *p* value of the factors were shown above the box. (c) Boxplots of external validation AUC under five disease categories in 16S genus data (dataset excluded IBD). Points represent the external validation AUCs, and colors represent the different disease categories. Kruskal–Wallis test was used for multiple-group comparisons and *p* value was shown at the top of the picture. Multiple pairwise Wilcoxon rank sum test comparisons were adjusted and *p* values were shown above the line segment. Box elements show the median and upper and lower quartiles. (d) Boxplots of external validation AUCs between mNGS species and 16S genus in intestinal disease which only included disease with all three data types, including CD, CRC and UC). Colors represent the different data types. Two sides Wilcoxon rank sum test was used and *p* values were shown above the picture. Box elements show the median and upper and lower quartiles.

Since the above analysis could be confounded by the disease category, we dissected the contributions of the two factors (i.e., data type and disease category) to the external validation AUCs using a two-way analysis of variance (ANOVA). Due to the imbalanced design of our data, i.e., the number of observations is different in different treatments, we found that both contributed significantly to the external AUCs (Figure 3a, *p* < 2.1e-6, ANOVA; Table S3) when first adjusting the data type and then the disease categories as covariables. However, we identified the disease category as the only significant contributor (Figure 3b, *p* < 2.1e-6, ANOVA; Table S3) when first adjusting the disease category and then the data type. These results implied that the disease category is a predominant determinant of reproducibility^88^, whereas the data type might be a significant factor only in certain disease categories.

We then reanalyzed the external validation results by considering the two factors at the same time. As shown in Figure 2c, when the data type was controlled, we confirmed the significantly higher external AUCs of the intestinal disease than that of the other three disease categories in mNGS-based species- and genus-level classifiers (Figure 2c, two subgraphs on the left; all pairwise Wilcoxon rank sum test adjusted *p* < 0.01; Kruskal–Wallis test: species *p* = 5.8e-09, genus *p* = 7.6e-08), but not in the 16S-based classifiers. Considering IBS is a functional bowel disorder and associated with stressful life events, we excluded it from intestinal disease and compared again. Then, we observed the intestinal disease was better than the mental disease (Figure 3c, pairwise Wilcoxon rank sum test adjust *p* = 0.0046). When controlling for the disease category, we observed significantly higher external AUCs of the mNGS-based species-level classifiers than the 16S-based genus-level classifiers only in the Intestinal disease (Figure 2d) especially for CD (Figure 3d). Furthermore, we did not observe any significant differences in the Metabolic, Mental and Autoimmune diseases (Figure 2c,d), and their generally low cross-cohort validation results regardless of the data type.

Together, our results suggested that mNGS-based classifiers could improve the cross-cohort validations, likely because they offered higher taxonomic resolutions. However, we only observed such improvements in intestinal diseases.

### Pooling of training cohorts substantially improves predictive performances in independent cohorts for non-intestinal diseases

**Figure 4.**
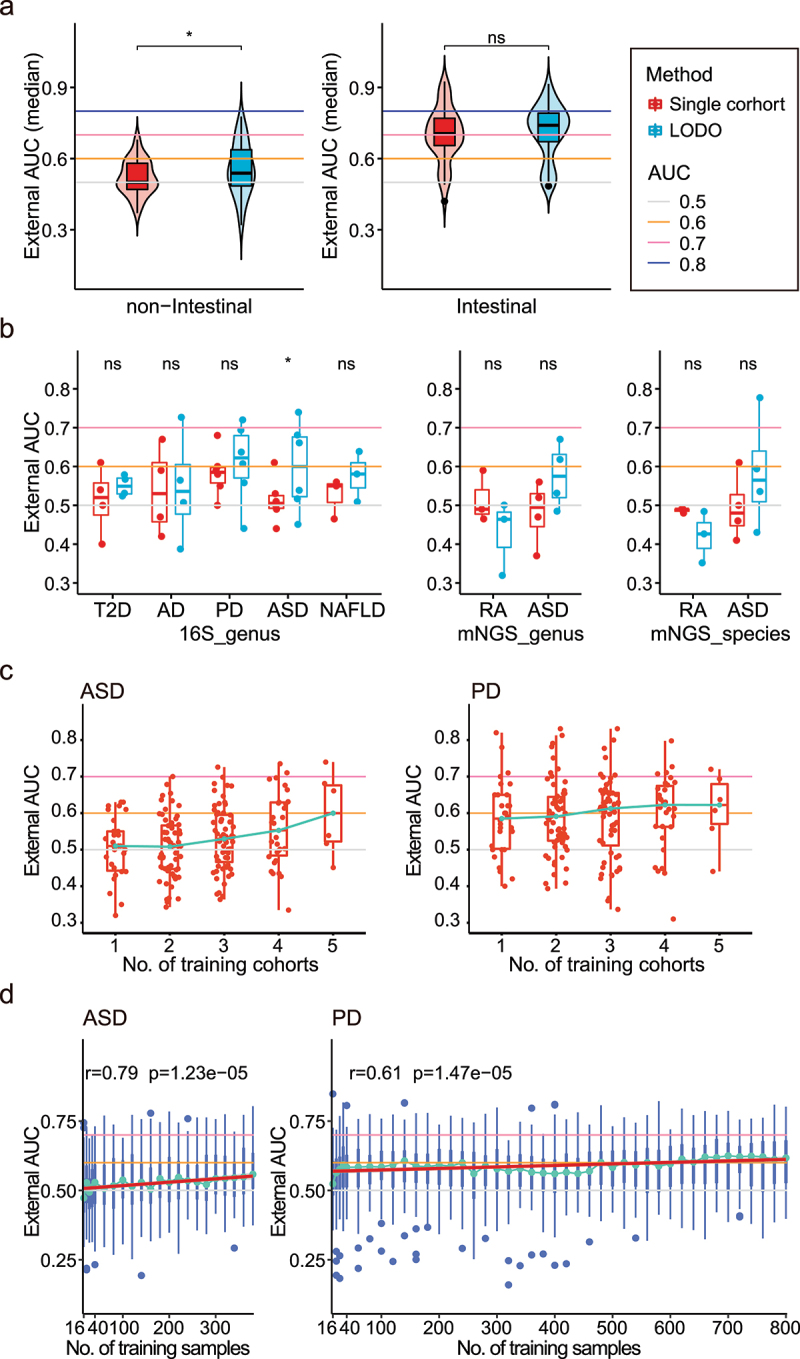
The improvement of the external validation in LODO and Cohort-Cumulation modeling. (a) Left: Comparation of median external validation AUCs between intra-cohort and LODO modeling method under non-intestinal diseases. Each point represents the median external AUC of each cohort (as testing dataset). Two-side paired Wilcoxon rank sum test was used for pairwise group comparisons. Right: Comparation of median external validation AUCs between intra-cohort and LODO modeling method under intestinal diseases. (b) Comparation of median external validation AUCs between intra-cohort and LODO modeling method per non-intestinal disease. Two-side paired Wilcoxon rank sum test was used for pairwise group comparisons. (c) External AUCs for the testing datasets at increasing numbers of training cohorts considered for the model (CCM). Non-intestinal diseases with more than or equal to 5 were shown here (including ASD and PD). The green line linked the median external AUC at each number of training datasets. (d) External AUCs for the LODO modeling at increasing numbers of samples considered for the training model (SCM). Non-intestinal diseases with more than or equal to 5 were shown here (including ASD and PD). The green line linked the median external AUC at each number of training datasets. The red line represents the linear regression model of the No. of training samples to median external AUC (Table S4), and Spearman correlation analysis was also carried out (the correlation coefficient and *p* value were shown at the top).

To overcome limitations of single-cohort classifiers, we performed three combined-cohort analyses by pooling samples from multiple cohorts for training and validated in independent cohorts (Figure 1a). First, a leave-one-dataset out (LODO) analysis was performed for each of 12 diseases (Figure 1b, Table S1) with ≥3 cohorts, which trained classifiers on *n*-1 datasets combined (where *n* was the number of all cohorts of a disease of interest), and validated them on the one left-out cohort, for each cohort in turn^89^. We observed increases of the median external AUCs for both the intestinal and non-intestinal diseases, respectively; among which, the increases for the latter were significant (Figure 4a, *p* = 0.027, paired Wilcoxon rank sum test). Closer examination on each non-intestinal disease indicated that the LODO analysis increased the external validation AUCs median for all diseases (T2D, AD, PD, ASD, and NAFLD) but one (RA; Figure 4b). For RA, the overall AUCs were likely affected by the much lower external validation results (0.38) between PRJ356102 and PRJ487636; in addition, samples from these cohorts accounted for, respectively, 53% and 38% of the total samples in the training set, which could lead to lower predictive accuracies in other cohorts. The increases varied from 1.13% to 13.27% among the diseases, although none reached statistical significance, except for the ASD 16S cohorts (Figure 4b, *p* < 0.031, paired Wilcoxon rank sum test). For each intestinal disease, we observed increases in the external validations AUCs for UC, CD with 16S data, and CRC with mNGS data, but not the IBS and Adenoma (Fig. S4A or Table S2). For IBS, the internal AUC of PRJ268708 (AUC = 0.97) was better than the other two cohorts (all AUCs<0.58), while the external AUC of PRJ268708 involved (median AUC = 0.46) was lower than the others (median AUC = 0.53), which may be due to over-fitting modeling of PRJ268708. For Adenoma, the external validation AUCs were generally low (0.59) indicating difficult to distinguish from health; nevertheless, our results were consistent with previous meta-analysis on Adenoma^39^. Overall, these results suggested pooling of training cohorts could improve predictive performances in independent cohorts for most diseases (9 out of 12, 75%).

We next tested whether adding more cohorts could continuously improve the external validation performances using a cohort-cumulation analysis for diseases with ≥5 cohorts, which trained models by randomly combining 2 to *n-*1 cohorts and validated them on the left-out ones (CCM). Four diseases met the criteria for such an analysis, including ASD, PD, CRC, and CD; among which, six 16S cohorts were available for ASD and PD, respectively, and seven and five mNGS cohorts were available for CRC and CD. For ASD and PD, we obtained continuously improved external validation AUC with the increase of the number of training datasets (Figure 4c). However, even when *n-*1 cohorts were combined as the training set (e.g., the same as the LODO analysis), we still observed very low external AUCs with medians of 0.6 and 0.62 for ASD and PD, respectively, indicating additional samples/cohorts may be required to further improve the predictive validation performance. For CRC and CD, we did not always observe the increased external AUCs with the increasing numbers of cohorts; however, we did obtain the highest predictive performance with the three cohorts and one cohort combined, respectively (0.77 and 0.89 for CRC and CD, respectively, Fig. S4B).

Together, our results suggested that combining samples from multiple cohorts as the training data, i.e., combined-cohort analyses, did improve the predictive performances in external validation, especially for the non-intestinal diseases.

### CD and CRC achieve high external validation with small sample sizes, whereas ASD and PD require more samples

To estimate the minimal number of required samples to train a classifier that can achieve high external validation performances, e.g., predictive AUCs ≥0.7, we performed a sample-cumulation analysis (one of combined-cohort analysis) on the diseases used in the cohort-cumulation analysis, for which we trained the classifiers by randomly selecting increasing number samples from the pool of *n-*1 cohorts combined, and tested them on the left-out cohort (Methods). For both ASD and PD, we observed an increasing trend in the external AUCs in a linear growth with the increasing number of training samples (Figure 4d; ASD: Spearman correlation coefficient *r* = 0.79, *p* = 1.23e − 05; PD: *r* = 0.61, *p* = 1.47 e − 05). Thus, applying a linear regression model to fit the relationships between the number of training samples and the external AUCs (median), we estimated that a total of 1,600 and 2,400 samples would be required for ASD and PD to achieve a median external AUC of 0.70 (95%CI: 0.62–0.78) and 0. 70 (95%CI: 0.64–0.76) (Table S4, both *p* < 0.0001, F-test).

Conversely, in both CRC and CD, we observed a rapid increase of the external AUCs at the very beginning of the sample-cumulation analysis, which quickly plateaued at 80 ~ 100 samples (Fig S4C); at this relatively small sample size, the external results were already very high, with 0.74 and 0.86 AUCs (~80 samples) for CRC and CD, respectively. In fact, with only~40 samples for the two diseases, we could obtain high AUCs of 0.73 and 0.86 for CRC and CD, respectively. After the plateau, the AUCs for CRC could be further improved with increasing number of samples, although at a much slower pace (Fig. S4C); however, the external AUCs for CD were not increased (Fig. S4C). These results were consistent with previous results^69^, and speculation that the direct interaction between the diseased site (i.e., the intestine) and the gut microbiota could greatly facilitate the classifier validation in independent cohorts.

### Cross-cohort marker consistency, measured by marker similarity index (MSI), showed similar trends to the modeling analysis

We also evaluated the consistencies of microbial markers (i.e., disease biomarkers) across cohorts of the same disease. The biomarkers often showed significant differences in their relative abundances between the case and control groups, and were targets for disease intervention and treatment. We identified the microbial markers using LEfSe (Linear discriminant analysis Effect Size; Methods), one popular method for disease marker identification in microbiome studies, and observed in general high consistencies among cohorts of intestinal diseases such as CRC, CD and IBD (Fig. S5). For example, markers showed excellent consistency among the seven mNGS cohorts for CRC (Figure S5); at the genus level, three genera were enriched in patients of all cohorts, including *Peptostreptococcus*, *Parvimonas*, *Porphyromonas*. At the species level, three species were enriched in patients of all cohorts, including *Peptostreptococcus stomati*, *Fusobacterium nucleatum*, *Gemella morbillorum*, followed by two disease-enriched species in six projects, including *Solobacterium moore* and *Porphyromonas asaccharolytica*. These results were consistent with previous studies^1,39,69^. Conversely, we observed low consistencies among cohorts of the non-intestinal diseases such as the ASD, RA and Adenoma (Figure S5). For example, microbial markers of ASD showed poor consistency across the six 16S cohorts (Figure S5): out of a total of 44 markers, most were either cohort-specific, or enriched/depleted in only two or three cohorts. The only genus, *Collinsella* that was identified as a marker in four cohorts, showed disease-enrichment in three projects, but health-enrichment in one other project (Fig. S5).

**Figure 5.**
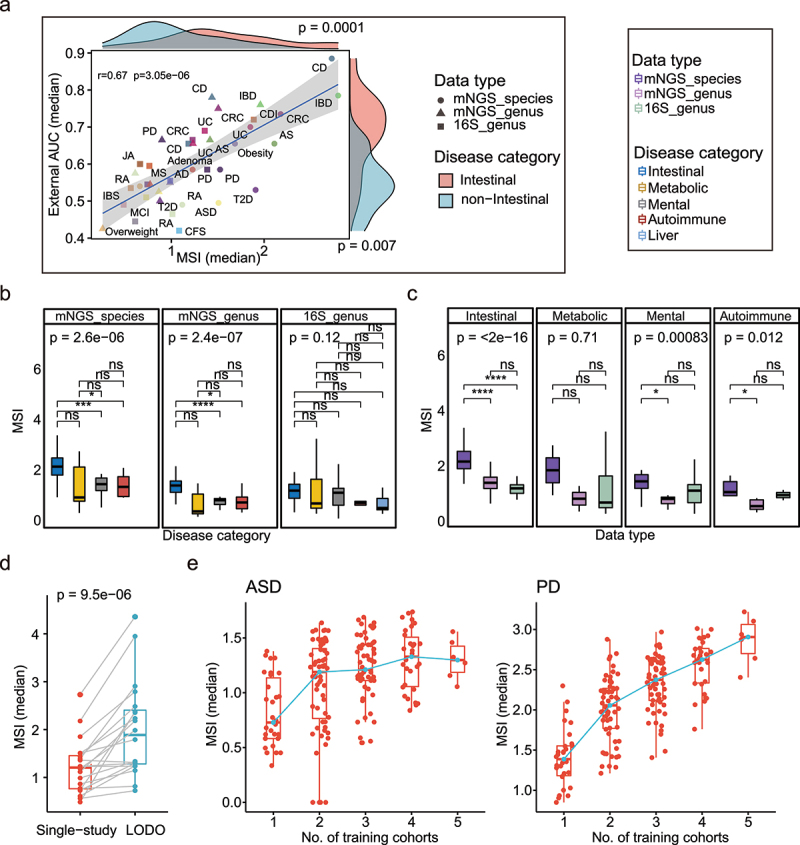
Association between external validation results and Marker Similarity Index (MSI) results. (a) Correlation between the median MSIs and external validation AUCs using intra-cohort modeling of each disease (Spearman r = 0.67 p = 3.05e − 06); the shape and color represent different data types and diseases. The x-axis value of each point represents the median of MSI when dataset under organization of intra-cohort modeling in each disease, and the y-axis value represents the median external validation AUC using intra-cohort modeling in each disease. The density distributions of x- and y-axis between intestinal and non-intestinal diseases were shown at the top and right. Two-side paired Wilcoxon rank sum test was used for pairwise group comparisons. (b) Boxplots of MSIs under different disease categories in each data type. The colors represent the different disease categories. Kruskal–Wallis test was used for multiple-group comparisons and *p* value was shown at the top of the picture. Multiple pairwise Wilcoxon rank sum test comparisons were adjusted and *p* values were shown above the line segment. Box elements show the median and upper and lower quartiles. (c) Boxplots of MSIs between different data types in each disease category. Dataset only included disease with all three data types. (d) Comparation of median MSIs when dataset under organization of intra-cohort and LODO modeling method in each disease. Two-side paired Wilcoxon rank sum test was used for pairwise group comparisons. (e) Median MSIs calculated from dataset under organization of CCM. The green line linked the median MSI at each number of training datasets. Non-intestinal diseases with more than or equal to 5 were shown here (including ASD and PD).

To quantify the cross-cohort marker consistency, we created a Marker Similarity Index (MSI) defined by the adjusted Euclidean distance between the Linear Discriminant Analysis (LDA) scores of the biomarkers from two cohorts (Methods). Higher (lower) MSI scores indicate higher (lower) cross-cohort marker consistencies. As expected, the median MSI scores significantly positively correlated with external validation AUCs of diseases (Figure 5a; Spearman Correlation Coefficient *r* = 0.67, *p* = 3.05e − 06), the same was true when training data organization under LODO modeling method (Fig. S6A; Spearman Correlation Coefficient *r* = 0.48, *p* = 3.11e − 02). Consistent with the modeling analysis, the MSIs of the intestinal diseases were significantly higher than the other three disease categories (Figure 5b left two and Fig. S6B, *p* < 0.05, Kruskal Wallis test); in addition, the MSIs at the species level were significantly higher than the genus-level (Figure 5c, all Kruskal Wallis test *p* < 0.05 except Metabolic disease category). Moreover, we observed significantly increased MSI scores when dataset organization under combined-cohort analysis, including both the LODO and cohort cumulative methods (Figure 5d,e, Fig. S6C).

*p < 0.05, **p < 0.01, ***p < 0.001, ****p < 0.0001

Of note, we obtained similar results using MSI scores calculated from other microbial marker identification tools, including ALDEx2 and MaAsLin2, which were recommended by recent two publications^90,91^ in which a total of 38 and 11 such methods were evaluated respectively. As shown in Fig. S7, we observed significant positive correlations between the external AUCs and the MSI scores calculated from the makers identified by ALDEx2 or MaAsLin2 (Fig. S7A); in fact, the MSI scores based on all the three marker identification methods, namely LEfSe, ALDEx2 and MaAsLin2 showed similar trend across disease categories and data types (Fig. S7BC), and had strong pairwise positive correlations (Fig. S7D) regardless of difference of them (Fig. S7E). However, we did obtain the highest correlation between the LEfSe-based MSIs and the external AUCs (*r* = 0.67 as comparing 0.43 and 0.46 for the other two tools, respectively). Nevertheless, our MSI calculation was robust regardless of the maker identification methods.

Together, our results indicated that microbial marker consistencies across cohorts were generally low for non-intestinal diseases, but could be significantly improved by the combined-cohort analysis, consistent with the modeling analyses above.

## Discussion

Due to its relevance in human diseases, the human gut microbiome has been increasingly used as biomarkers for noninvasive disease prescreening, and targets for disease intervention. However, because the gut microbiome can be significantly affected by many factors, controversies exist with regard to the reproducibility of gut dysbiosis in different cohorts. In this study, we comprehensively evaluated the reproducibility of gut microbiome as diagnostic prescreening tools in 83 disease-control cohorts for 20 diseases. We built machine learning classifiers using taxonomic species- and/or genus-level taxonomic relative abundances of the gut microbes, and performed intra-cohort, cross-cohort and combined-cohort predictive validations for each of the diseases. We focused on the external validations, i.e., applying the classifiers in independent cohorts, and identified three significant influential factors (i.e., the determinants), namely the disease category, the data type, and the sample size. First, single-cohort classifiers of all but the intestinal diseases in general failed to accurately predict diseases in cross-cohort validation analysis, with averaging AUCs of 0.64 (0.73 for the intestinal diseases, 0.54 for the non-intestinal diseases). Second, mNGS data that were known to provide higher taxonomic resolution than the 16S amplicon data, could significantly improve external validation performance, but only for the intestinal diseases. Last, using increased number of samples as the training data, e.g., by pooling samples from multiple cohorts, could substantially improve the predictive performances of the resulting classifiers in external validations, especially for the non-intestinal diseases. However, to reach a practical AUC of 0.70, much larger numbers of samples would be required for the non-intestinal diseases such as ASD and PD. Our results were consistent with previous studies that reported high cross-cohort validation results for CRC^39,69^ and CD^83^, low external AUCs in T2D^69^ and Adenoma^39^, and supported that the combined-cohort analyses including LODO and cohort-cumulation could improve external AUCs^39,69,70^. We also analyzed the consistency of disease biomarkers across cohorts of the same diseases, and found essentially the same trends (i.e., markers in general did not agree across cohorts with the except for intestinal diseases) and determinants (i.e., disease category and sample size). Overall, our results support the use of gut microbiome as independent, cross-cohort diagnostic tools for only handful intestinal diseases.

The gut microbiome is known to be significantly affected by many factors, including diseases^39,92^, diet^56^, drugs^57,58,63^, seasonal changes^93^, regional differences^59^, genetic backgrounds^94–96^, sample preprocessing and data analysis methods^60–62^. We argue that factors that have consistent gut microbial signatures and significantly affect the gut microbiota, can greatly promote cross-cohort predictive validation of the gut microbiome-derived classifiers. One such factor is the intestinal diseases such as CRC and CD (or IBD). These diseases are associated with significant and global changes of the intestine that render direct and significant effects on the gut microbiota, and mask the effects of other environmental and technical factors; in addition, they also have consistent gut microbiome signatures such as the *P. stomatis*, *F. nucleatum*, *G. morbillorum* in the CRC, and *Ruminococcus gnavus*, and *Veillonella* species in the CD. Consequently, classifiers based on relatively small sizes of samples (~less than 100) validated well in independent cohorts (Fig. S3BTable S2). This was more evident in the sample-cumulation analysis that as few as 40 samples randomly selected from multiple cohorts could achieve high predictive performance of 0.73 and 0.86 AUCs for CRC and CD, respectively (Fig. S4C). Part of the reason was that sampling from multiple cohorts could help combine common characteristics between multiple cohorts, and generated classifiers with improved generalization ability. This line of reasoning correctly predicted the low cross-cohort validation performance of Adenoma (Fig. S3A), which did not cause global changes of the intestine, and IBS, which did not significantly affect the gut microbiota when dormant (Fig. S3A). The other such factor includes drugs such as proton pump inhibitors (PPI) and metformin that have distinctive gut microbiome signatures. In the present study, we did not specifically analyze the impacts of drug usage on cross-cohort validations, because treatment information was largely unavailable for most cohorts due to ethical reasons. However, there were plenty of related discussions in the literature^12,58,65,66^. These drugs often dominated the alterations in gut microbiome whose signatures showed cross-cohort^12,42,66^, and even cross-disease consistencies^58^. For example, PPI is commonly used to treat liver diseases^97^ and multiple types of cancers^98^, and causes significant increase of oral bacterial species in the gut microbiota, especially those belonging to the genera of *Veillonella* and *Streptococcus*^58,65,66,99^.

Conversely, factors that do not have consistent gut microbiota signatures often undermine the cross-cohort predictive performance, such as diet, age, BMI, sample preprocessing, and batch effects. These factors are either too complicated to quantify in general (e.g., diet) or have inconclusive effects on the gut microbiota according to the literature^59,69^. In addition, disease definition, and diagnostic criteria could be different for complex diseases such as ASD, PD in different cohorts; for diseases with subtypes, the relative proportions of the subtypes could also be different across cohorts. These will further undermine the cross-cohort validation results. Unfortunately, these detailed meta-data are often unavailable for most cohorts^100^. Thus, although we had adjusted within and cross-cohort confounding factors, our results represented the lower limits of the cross-cohort validation performance of gut microbiome-based disease classifiers, which could be certainly improved if above-discussed factors were properly recorded and controlled.

Despite being the largest meta-analysis on disease-related gut microbiomes to date, our study had the following limitations. First, we were only able to include a small fraction of human gut microbiota research published. Especially for combined-cohort analysis, only few diseases were available. This was in part due to the lack of reporting guidelines for human gut microbiome researches that were only publicly available in late 2021^101^, and central repositories to enforce the guidelines and accommodate essential meta-data such as age, gender, BMI, health and disease statuses. Consequently, over two-thirds of the human gut microbiome samples deposited to general-purpose sequence archives such as NCBI SRA (Sequence Read Archive)^102^ and ENA (European Nucleotide Archive)^103^ lacked the essential information such as age, gender or BMI^100^. Microbiome-centric databases including MGnify^104^, MG-RAST^105^, and gcMeta^106^ only partly solved the issues but did not enforce characteristics essential for understanding human health and diseases. Recently, databases and resources with consistently analyzed human gut microbiomes and manually curated meta-data were made available by several research groups, including HumanMetagenomeDB^107^, GMrepo^68,100^ and curatedMetagenomicData^108^. These would significantly promote meta-analysis of disease-related human gut microbiomes, but systematic efforts are still needed in the future. Second, we were only able to control limited numbers of known confounding factors for the intra-cohort and cross-cohort analyses, due to lack of metadata in most cohorts (Table S1). Third, we were not able to reevaluate the excellent validation results in independent cohorts reported for several diseases, including Hepatocellular Carcinoma (HCC)^109^, and ASD^84^, due to unavailable discovery sequencing data and/or corresponding meta-data (Table S5). Further efforts would be required to include more datasets and diseases. Finally, more advanced data pre-processing methods should be tested thoroughly and consequently apply to microbiome analysis. For example, the taxonomical composition from a metagenomic sample is often sparse and compositional^110^, with the taxa being phylogenetically and/or functionally related^111^. Conventional linear regression model may not perform well and require more computational power when the predictor variables (taxa) are high dimensional and highly related. However, the Lasso algorithm with the penalty term and RF outperformed many others such as SVM and deep learning when applied to human disease stratification, likely because of its excellent ability to handle small datasets^37,112^ (e.g., with 50 samples or less). Recently, researchers proposed to aggregate the sparse signals based on either phylogenetic relatedness^111,113,114^, or functional similarities^115^, and some could perform better than Lasso in terms of feature selection although their applicability in disease stratification is yet to be tested. Regardless, these data-processing methods should be systemically evaluated in the near future. In addition, we encourage more comprehensive guidelines of microbiome public resource and analysis, which promotes meta-analyses to get reproducible findings.

In conclusion, we systematically evaluated the reproducibility of gut microbiome as disease markers and diagnostic prescreening tools for 20 diseases, and identified their dormant factors. We supported strongly the feasibility for gut microbiome as independent, cross-cohort diagnostic tools for several intestinal diseases, and recommended strategies to improve the cross-cohort predictive performances for non-intestinal diseases.

## Material & methods

### Study collection

We first compiled a comprehensive list of human disease related case–control studies on gut metagenome by searching in public databases including MGnify (https://www.ebi.ac.uk/metagenomics/)^104^, NCBI Sequence Read Archive (SRA) (https://www.ncbi.nlm.nih.gov/sra)^102^ and GMrepo (a curated database of gut human metagenomes; https://gmrepo.humangut.info)^68,100^, which included 361 case–control studies on 134 different diseases (Table S1, the run-level data included 34,702 cases and 45,429 controls). Studies that contained more than two different diseases were only counted once. We excluded projects that had incomplete disease phenotype metadata, or contained<15 samples in the case or control groups (Table S1). We synthesized the supplementary samples by using an over-sampling technique known as SMOTE (Synthetic Minority Oversampling TEchnique) when the ratio between case and control was out of balance^116^ (see details below).

We divided the qualified projects into two subcategories according to their sequencing strategies, namely 16S ribosomal RNA gene amplicon sequencing (16S) and shotgun metagenomic next-generation sequencing (mNGS). In each subcategory, we also excluded diseases that had only one study, in order to perform cross-study/cohort comparisons. In the end, we retained in total 69 studies on 20 diseases, including 41 16S studies on 15 diseases (sample-level: 3,573 cases, 2,090 controls), and 28 mNGS studies on 12 diseases (sample-level: 2,411 cases, 1,634 controls) (Table S1 all_models_data, Figure 1b). Among these, seven diseases had both multiple 16S and mNGS studies (Table S1, Figure 1b).

We divided the 20 diseases into five disease categories according to the Medical Subject Headings (MeSH, https://meshb.nlm.nih.gov/) database and Human Disease Ontology (DO) database^77^, including Intestinal, Autoimmune, Metabolic, Mental and Liver disease types. Intestinal diseases included those associated with the intestinal tract, whereas the Mental diseases here represent Mental and Nervous System disorders.

### Sequencing data processing and taxonomic annotation

Raw sequencing data were downloaded from the NCBI SRA^102^ or European Nucleotide Archive (ENA)^117^. Trimmomatic^118^ was used to trim the reads to remove sequencing vectors and low-quality bases; reads shorter than 50 bps after trimming were also removed. The remaining reads were referred to as ‘clean reads’.

For mNGS data, putative human reads were identified by aligning the ‘clean reads’ to the human reference genome (hg19) using Bowtie2^119^ with default parameters, and removed from subsequent analysis. Multiple sequencing runs corresponding to the same sample were merged. MetaPhlAn2^120^ (with default parameters) was used for taxonomic profiling and to calculate the relative abundances of recognizable taxa at various clades from phylum to species. In the end, we retained the relative abundance information at genus and species levels for each sample for downstream analysis.

For 16S data, QIIME2 version 2021.2 pipeline^121^ was used. Raw data were denoised to amplicon sequence variants (ASVs) tables by DADA2 version 1.18.0^122^. Taxonomic assignment of the individual dataset was classified against the Greengenes database version 13.8^123^. Genus-level relative abundance results were retained for subsequent analyses. We also annotated the 16S data for three diseases which had more cohorts (including ASD, T2D, PD) for taxonomy information using the Silva database (https://www.arb-silva.de, version 138) (Table S2) and found similar model performance (i.e., both the internal and external AUCs) to the Greengenes-based annotations (Fig. S8A). Thus, the taxon abundance data classified against the Greengenes database were used in the subsequent analyses.

Then, samples with two taxa or fewer were also removed from further analyses. To avoid noises caused by low abundant taxa, those with relative abundance<0.001 across all samples were filtered.

### Removal of confounders and batch effects

Before disease marker identification and disease prediction modeling, we identified confounding factors for each cohort and subsequently removed their effects on the taxonomic relative abundance profiles. To do so, we checked all available factors in the metadata such as age, gender, body mass indexes (BMI), disease stage and geography, and tested whether they were significantly different between the case and control groups of a cohort. We used the Fisher’s exact test for qualitative variables (including age, BMI), and the non-parametric Wilcoxon rank sum test for quantitative variables (including gender, disease stage and geography). Factors with *p* values<0.05 were adjusted in the study using *removeBatchEffect* function implemented in the ‘limma’ R package^124^ (v.3.46.0, significant qualitative and quantitative variables as *covariates* and *batch* respectively, others with default).

To facilitate cross-study/cohort comparison, we also removed batch effects using the *adjust_batch* function implemented in the ‘MMUPHin’ R package^125^ (v.1.4.2) by using project id and co-confounders as the controlling factors.

Subsequent analyses were performed on the relative abundance data after either the removal of confounders (for LEfSe) or the removal of both confounders and batch effects (for intra-cohort modeling and combined-cohort modeling).

### Modeling data preprocessing, machine learning modeling, and performance evaluation

The ‘SIAMCAT’ R package v.1.9.0 (https://bioconductor.org/packages/SIAMCAT)^83^ was used to build disease-stratification classifiers (or models). Briefly, the following parameters were used as recommended by the authors: (1) use default feature cutoff of 0.001 to filter lowly abundant taxa in *filter.features* function; (2) set *norm.method = ‘log.std’* and default *norm.param* (*log.n0* = 1e-06, *sd.min.q* = 0.1) to normalize filtered relative abundances in *normalize.features* function, which means log-transforms (after addition of pseudocounts) and applying z-score standardization; (3) the *num.folds* and *num.resample* parameters in *create.data.split* function were adjusted for different data combination methods such as intra-cohort, combined-cohort modeling.

Predictions were performed using the *make.predictions* function. Prediction performances were evaluated by the area under the receiver operating characteristic curve (AUROC or AUC) scores using the ‘pROC’ R package (implemented in the *evaluate.predictions* function in ‘SIAMCAT’ package).

For comparison, we also calculated two other performance measurements including the area under the precision-recall curve (AUC-PR) and Mathews Correlation Coefficient (MCC). The AUC-PR was calculated by the *evaluate.predictions* function with default parameters implemented in the ‘SIAMCAT’ R package. The MCC was calculated using the *mcc* function with default parameters implanted in the ‘mltools’ (v.0.3.5) R package (https://github.com/ben519/mltools), which also calculated the true positive (TP), false positive (FP), true negative (TN) and false negative (FN) rates.

### Selection of machine learning algorithm for disease modeling

To select the best machine learning algorithm for disease modeling, we compared four such methods including Elastic Net (Enet)^78^, Lasso^79^, Random Forest (RF)^80^ and Ridge Regression (Ridge)^81^ implemented in SIAMCAT. The *method* parameter (*‘lasso’, ‘enet’, ‘ridge’, ‘randomForest’*) in *train.model* function controlled the choice of the machine learning algorithm.

### Dealing with imbalanced cohorts

An imbalanced cohort refers to those with significantly more cases (e.g., three times in this paper) than the controls, and vice versa. Modeling on unbalanced cohorts often leads to biased classification toward the majority class samples^126^. In this study, an over-sampling method called SMOTE^116^ was used to increase the rare group samples. This method was based on a k-nearest neighbor (KNN) algorithm, and implemented in the ‘smotefamily’ (v.1.3.1) R package *SMOTE* function (https://CRAN.R-project.org/package=smotefamily). We took *dup_size* parameter to be the maximum between three and half ratio of large group number to small one, which represents the desired times of synthetic minority instances over the original number of majority instances. And other parameters with default. We compared the AUCs (internal and external) calculated by the microbiome data processed by SMOTE before and after (Fig. S8B, Table S1–2). We found AUCs calculated by data processed after SMOTE were significantly higher than that original data (Fig. S8B, internal: *p* = 0.002, external: *p* = 0.0067, paired Wilcoxon rank sum test).

### Internal validation versus external validation

Internal validation was referred to as training on the part of the dataset and testing on the left part from the same cohort. On the other hand, external validation was referred to as training on one dataset and testing on independent cohort(s) (also referred as cross-cohort validation). To make full use of the samples and get strict cross-validation results with best hyper parameters, a nested cross-validation strategy was used as recommended by the authors of the ‘SIAMCAT’ R package^83^. For example, nested 5 folds cross-validation was: first, the dataset was randomly split into 5 outer folds, then 4 folds samples were combined to train and the left 1 fold to test, and then performed a grid search on the 4 outer folds training dataset through inner 5-fold cross-validation in order to find the best hyperparameters^85^. We referred the training models as single-cohort models. The model performances were measured by AUC scores. Notably, in external validation, the absent features which presented in the training set but not in the testing set were supplemented by 0.

### Intra-cohort modeling and validation

The intra-cohort modeling (i.e., single-cohort classifiers) was carried out for each cohort using five-fold three times cross-validation. The model was built using the *train_model* function (*num.folds = 5, num.resample = 3* in *create.data.split* function) implemented in the ‘SIAMCAT’ R package^83^. The prediction (or validation) used *make.predictions* and *evaluate.predictions* functions. The intra-cohort validation was internal validation.

### Combined-cohort modeling and validation

In this study, three combined-cohort modeling and validation strategies were performed for diseases with required numbers of available cohorts, including the leave-one-dataset out (LODO) analysis, cohort-cumulation modeling (CCM), and sample-cumulation modeling (SCM).

First, a LODO analysis^39^ was applied to diseases with ≥3 cohorts, which trained classifiers on *n-1* datasets combined, and validated them on the one left-out cohort, for each cohort in turn^89^. Here, the *n* referred the number of cohorts for a given disease. The LODO analysis examines whether combining multiple cohorts for modeling training can improve the predictive performance of the resulting classifiers.

Second, a cohort-cumulation modeling (CCM) was applied to diseases with ≥5 cohorts, which randomly selected and combined a certain number of cohorts for training, and tested the remaining cohorts in the same disease. The diseases that met this requirement included ASD, AD, CRC and CD. The CCM analysis examines whether the model performance can be improved with the increasing number of training cohorts.

Last, a sample-cumulation modeling (SCM) was also applied to diseases with ≥5 cohorts, which randomly combined increasing numbers of samples selected from the LODO training dataset and then testing on the remaining cohort of the same disease. The ratio of case to control when selecting training samples was set to 1:1. We showed the increasing number from 16 to 40 with interval of 6 and from 60 to maximum with interval of 20. The SCM analysis examines whether the model performance increases with the increasing number of samples in modeling training.

For the above combined-cohort modeling, we used ten folds three times repeated cross-validation (*num.folds = 10, num.resample = 3* in the *create.data.split* function) and only noted external (cross-cohort) validation AUCs. For CD and CRC which were mNGS data type, species level data was used to model for CCM and SCM. Otherwise, 16S datasets genus level was used for modeling.

The list of diseases used for the above analyses could be found in Table S1.

### Disease marker identification using linear discriminant analysis effect size (LEfSe)

Disease marker taxa were identified using LEfSe analysis^127^ implemented in the ‘microbiomeMarker’ R package *run.lefse* function (v.1.0.2, downloaded from https://github.com/yiluheihei/microbiomeMarker)^128^. The effect size score output, i.e., the linear discriminant analysis (LDA) score, can reflect the extent of the differences, with the larger value indicating the more significant differences.

Taxa with LDA scores ≥2 were considered markers. In this study, we assigned a plus (minus) sign to the score to indicate the corresponding marker was enriched in the case (control) group.

### Measuring the similarity of disease markers between cohorts of the same disease

To measure the marker similarity between two cohorts, a Marker Similarity Index (MSI) was created. The MSI was calculated using the following equation by taking the two corresponding LDA score vectors of the markers as inputs. Let A and B be the two cohorts, and the vectors $M^{A}=\left(m_{1}^{a},\ldots ,m_{p}^{a}\right)$ and $M^{B}=\left(m_{1}^{b},\ldots ,m_{p}^{b}\right)$ be the LDA scores of their markers. The two vectors should be equal in size and aligned according to the p markers in A (considering $MSI_{A\to B}$). Therefore, markers that are not in A will be removed from B, whereas those that are not in B will be supplemented with LDA scores of zero. The final MSI score will be calculated as the Euclidean distance of two vectors. When A only had one or no marker, the $\;MSI_{A\to B}$ is defined as 0.

$$MSI_{A\to B}=\frac{1}{\frac{dist\left(M^{A},M^{B}\right)}{p}}=\frac{p}{\sqrt{\sum_{i=1}^{p}{\left(m_{i}^{a}-m_{i}^{b}\right)}^{2}}}.$$

Thus, the MSI is asymmetrical: $MSI_{A\to B}$ and $MSI_{B\to A}$ can have different values considering different references. $MSI_{A\to B}$ regards A cohort as the reference, which can be analogous to training sets in cross-cohort validations.

To examine the effects of LDA cutoffs on the MSI, we calculated the MSIs with different LDA cutoffs ranging from 0 to 4. We found that the MSIs were stable between 0 and 2 (Fig. S9A) and then decreased rapidly after LDA>2 because fewer markers were retained (Fig. S9B). We randomly chose the MSI values calculated from several LDA cutoffs between 0 and 2, and found significant positive correlations between MSIs and external AUCs (Fig. S9C). Thus, the LDA cutoffs do not affect our main conclusion. In this study, taxa were considered as markers with LDA scores≥2.

For comparison, we also examined if our MSI calculation was robust against different marker identification methods. We selected two such methods, including ‘ALDEx2’ (implemented in the ‘ALDEx2’ R package (v.1.26.0)), and ‘MaAsLin2’ (implemented in the ‘MaAsLin2’ R package (v.1.26.0)), which were recommended by two recent publications in which a total of 38 and 11 such methods were evaluated^90,91^. For ‘ALDEx2’ and ‘MaAsLin2’, we selected features with Benjamini-Hochberg (BH) FDR-corrected p-value<0.1 as markers and used the output effect (the median of the ratio of the between group difference and the larger of the variance within groups) and coef (the coefficient from the fit model) values to calculate the MSIs, respectively. As mentioned before, we obtained the highest correlation between the LEfSe-based MSIs and the external AUCs, we used the LEfSe-based MSI calculations in the subsequent analyses.

### Comparing model performance with or without feature selection

To avoid over-fitting issues caused by label leakage, a nested feature selection strategy was used as recommended by *Wirbel* et al.^83^. Briefly, a specific number of top features were selected during cross validation for the training set by calculating the single feature absolute AUC and sorting the resulting AUCs decreasingly. The parameters *perform.fs* = TRUE, *param.fs*=list(*thres.fs*=num, *method.fs*=“AUC”, *direction*=‘absolute’) were set in *train_model* function, where num was the number of top features (11, 15, 20, 25, 30, 35, 40). Then, we compared the validation AUCs between modeling by a certain number of top features and all features to determine whether feature selection procedure should be used for this study. We used five diseases to perform the feature selection analysis, including AD, ASD, CD, CRC, and PD.

Please note, we only did the feature selection to test the performance of the classifiers and compare to that of the all-feature classifiers. In the end, we did not use feature selection because the all-feature classifiers performed the best in cross-cohort validation

### Comparing model performance with or without functional annotation of mNGS samples

To explore whether the pathway data can help improve the modeling performance, we collected the microbiome taxon and pathway abundance data from the curatedMetagenomicData (https://waldronlab.io/curatedMetagenomicData/) R package^108^, because the GMrepo v2 database did not include functional annotations. We retained four diseases that had at least three cohorts in curatedMetagenomicData, including Adenoma, IBD, T1D and T2D (Fig. S10A). We found that both the internal (Fig. S10B) and external (Fig. S10C) AUCs were comparable between the taxon-based models and those used the combination of the taxon and the pathway data (*p* > 0.05; Wilcoxon Rank Sum Test; see also Table S7 for details), suggesting that adding functional profile did not significantly improve the model performance. The results were similar to *Wirbel* et al. (Figure 2ab in corresponding reference)^39^ and *Thomas* et al. (Figure 3ad in corresponding reference)^129^ on CRC. Thus, we used the taxon abundance to build models in the subsequent analyses.

### Statistics and other bioinformatics analyses

All processed data, if not otherwise stated, were loaded into R (version 4.1.2, https://www.r-project.org/), analyzed and visualized. Wilcoxon rank sum test and corresponding pair test for pairwise data were used for two-group comparisons, while the Kruskal–Wallis test was used for multiple-group comparisons by using ‘ggpubr’ (v.0.4.0) package (https://github.com/kassambara/ggpubr) in *stat_compare_means* function with default parameters. The Wilcoxon rank sum test *p* values were corrected using ‘ggpubr’ package *compare_means* function with default parameters when performing multiple hypothesis tests. The Spearman correlation test was used for correlation analysis. All tests of significance were two-sided, and *p-value* < 0.05 (when the two groups were compared) or corrected *p-value* < 0.05 (when comparing multiple groups) was considered statistically significant.

## Supplementary Material

Supplemental Material

## Data Availability

The processed data and codes that support the findings of this study are available in GitHub repository at https://github.com/whchenlab/GMModels. These data were derived from the following resources available in the public domain: NCBI (https://www.ncbi.nlm.nih.gov/sra), ENA (https://www.ebi.ac.uk/ena/browser/), MGnify (https://www.ebi.ac.uk/metagenomics/), GMrepo v2 (https://gmrepo.humangut.info), and the accession codes were in TableS1.
