# Supplementary material for: Performance of Gut Microbiome as an Independent Diagnostic Tool for 20 Diseases: Cross-Cohort Validation of Machine-Learning Classifiers
Source: Gut Microbes. 2023 May 4;15(1):2205386. doi: 10.1080/19490976.2023.2205386 (PMC10161951; doi:10.1080/19490976.2023.2205386)
Supplement: Supplemental Material [file KGMI_A_2205386_SM1540.zip › Supplementary Material/Supplementary Figures and descriptions.pdf]

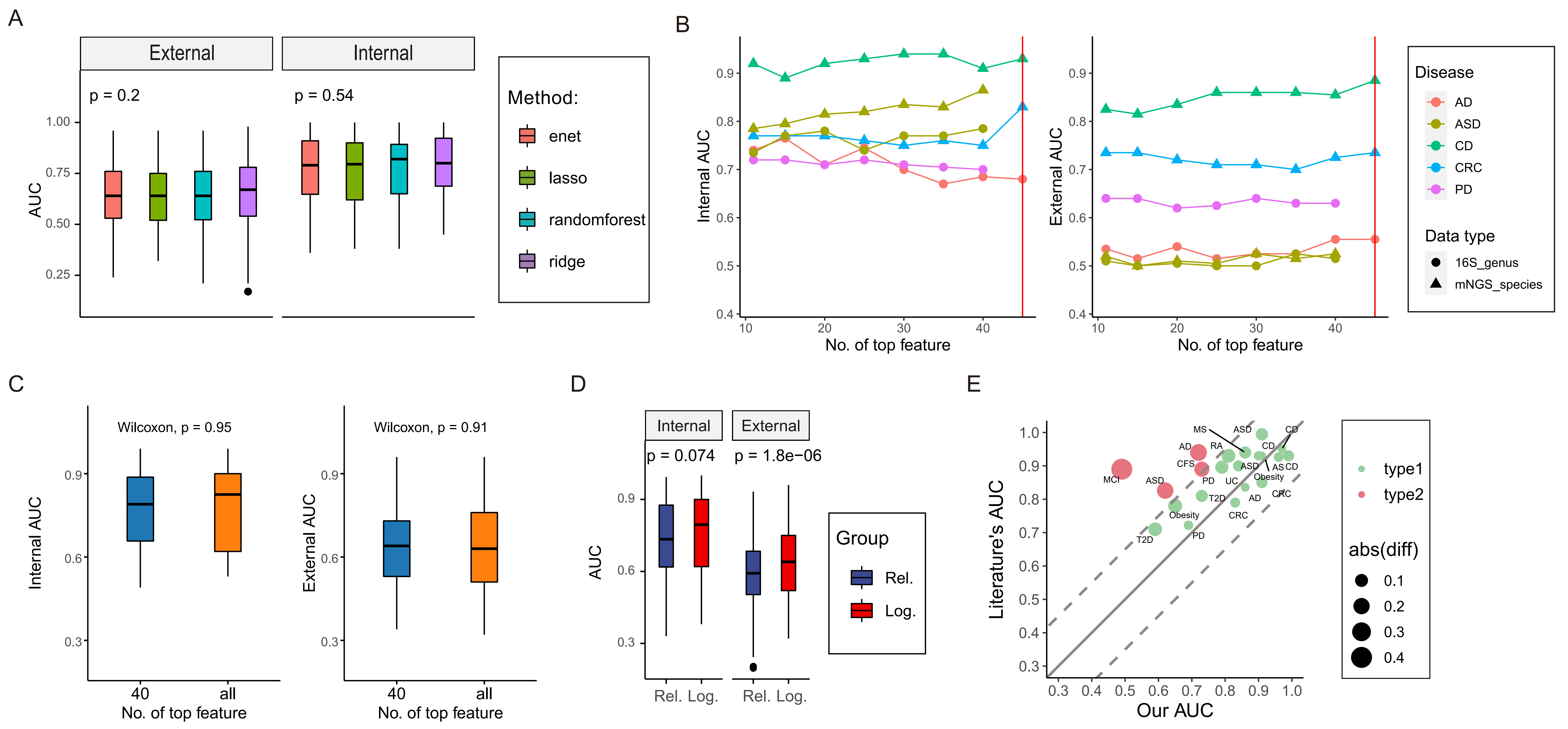

A
External
Internal
p = 0.2
p = 0.54
AUC
Method:
enet
lasso
randomforest
ridge
B
Internal AUC
External AUC
No. of top feature
No. of top feature
Disease
AD
ASD
CD
CRC
PD
Data type
16S_genus
mNGS_species
C
Wilcoxon, p = 0.95
Wilcoxon, p = 0.91
Internal AUC
External AUC
40
all
No. of top feature
No. of top feature
D
Internal
External
p = 0.074
p = 1.8e−06
AUC
Rel. Log.
Rel. Log.
Group
Rel.
Log.
E
Literature's AUC
Our AUC
type1
type2
abs(diff)
0.1
0.2
0.3
0.4

Supplementary Figure 1. **Evaluation of different machine learning algorithms and feature selection on performance of disease stratification classifiers**.

A, Boxplots comparing four different algorithms (Enet, Lasso, Random Forest and Ridge) in internal (left) and external (right) validation AUCs. Box elements show the median and upper and lower quartiles. Kruskal-Wallis test was used for multiple-group comparisons. **B,** The internal (right) and external (left) validation AUCs with the increasing top features selected in diseases with cohort size more than 4 (including AD, ASD, CD, CRC, PD); The number of features increased by 5 from 15 to 40. The last x coordinate is modeling with all features, marked by the red line. **C,** Comparation of internal (right) and external (left) validation AUCs between 40 top and all features. Two sides Wilcoxon rank sum test was used for pairwise between-group comparisons. **D,** Comparation of AUCs (internal and external) before (Rel) and after (Log) logarithmical transformation of the relative abundances. **E,** Comparation of internal AUCs between the highest of our four modeling methods and the corresponding results reported by the literature. The size of the circle represents the absolute value of the difference (abs(diff)). Dashed and solid lines were $y = x \pm 0.15$ and $y = x$. Type1 and type2 represent the abs(diff) less than and greater than 0.15, respectively. See Table S5 for a list of the literature and the AUCs.

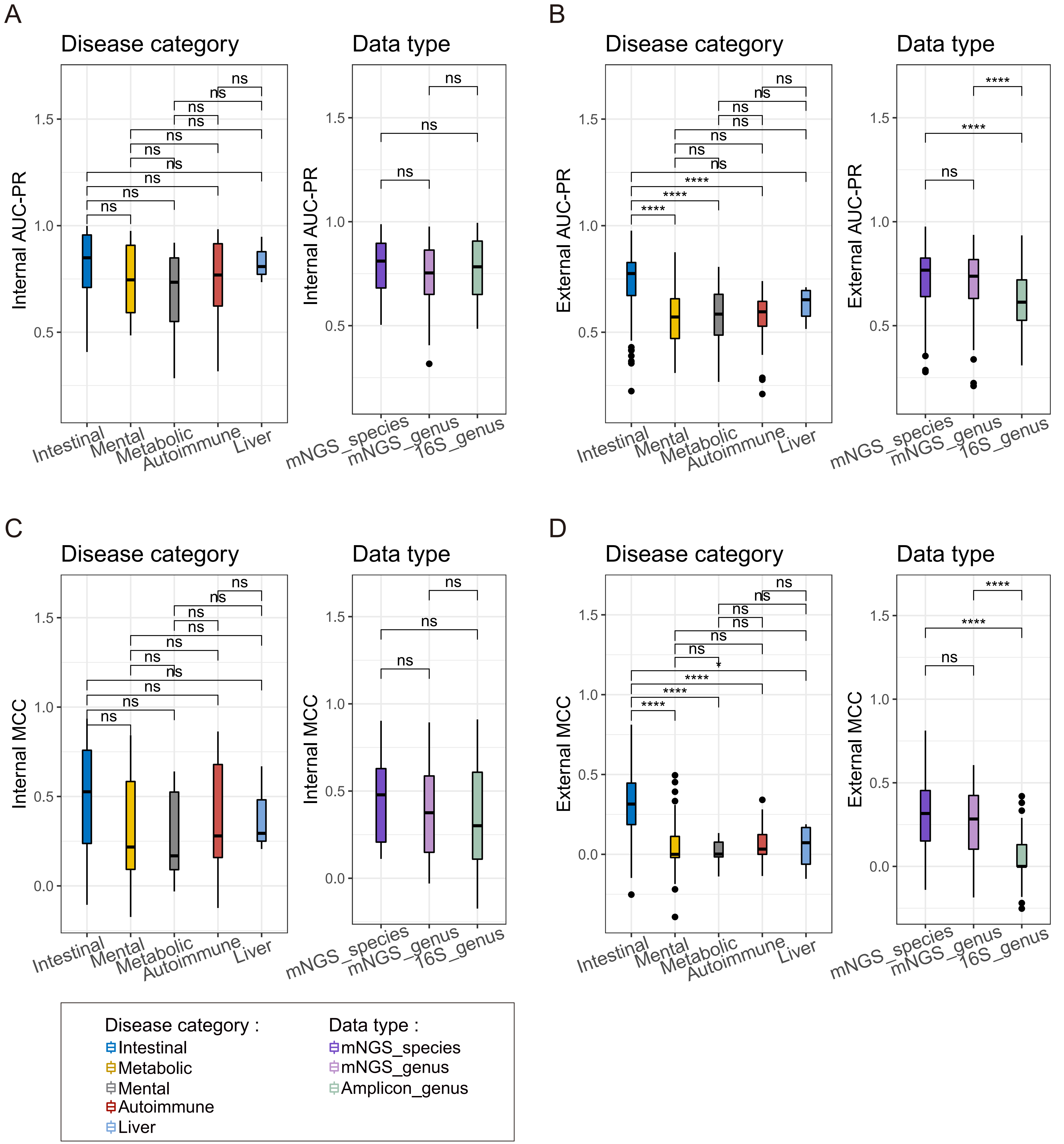
A
B
C
D
Disease category
Data type
Internal AUC-PR
External AUC-PR
Internal MCC
External MCC
ns
****
*
Intestinal
Mental
Metabolic
Autoimmune
Liver
mNGS_species
mNGS_genus
16S_genus
Disease category :
Intestinal
Metabolic
Mental
Autoimmune
Liver
Data type :
mNGS_species
mNGS_genus
Amplicon_genus

Supplementary Figure 2. **Comparation of the performance of intra-cohort classifiers on internal and external validation AUC-PR and MCC between different disease categories and data types.**

A, Left: Comparation of internal validation AUC-PR between different disease categories; Right: Comparation of internal validation AUC-PR between different data types. Multiple adjusted two sides Wilcoxon rank sum test was used for pairwise group comparisons. **B**, Left: Comparation of external validation AUC-PR between different disease categories; Right: Comparation of external validation AUC-PR between different data types. **C**, Left: Comparation of internal validation MCC between different disease categories; Right: Comparation of internal validation MCC between different data types. **D**, Left: Comparation of external validation MCC between different disease categories; Right: Comparation of external validation MCC between different data types. *$p < 0.05$, **$p < 0.01$, ***$p < 0.001$, ****$p < 0.0001$; pair-wise Wilcoxon Rank Sum test.

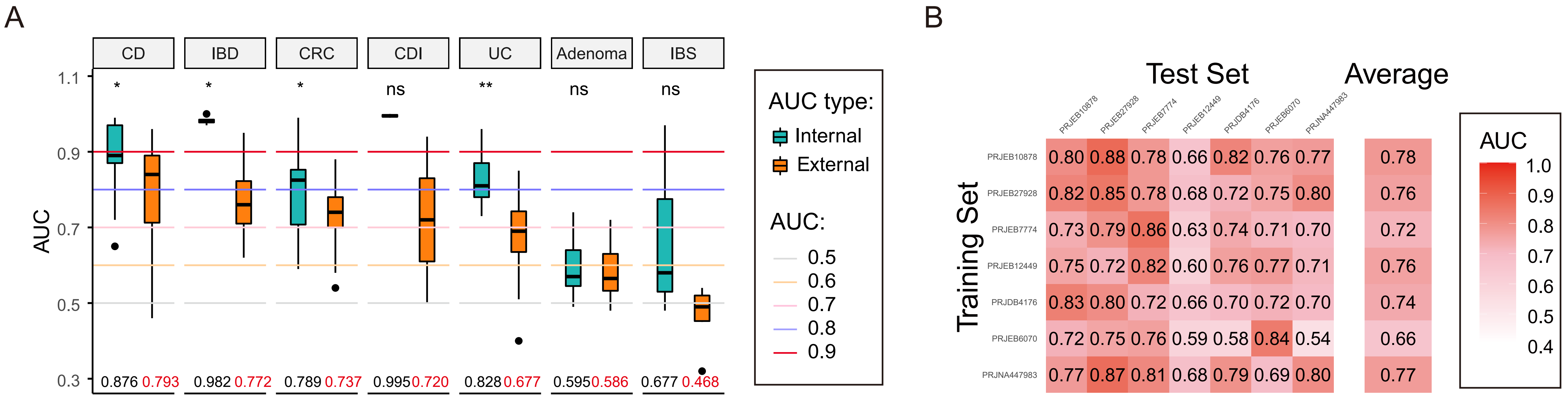

A
CD
IBD
CRC
CDI
UC
Adenoma
IBS
AUC
1.1
0.9
0.7
0.5
0.3
*
*
*
ns
**
ns
ns
0.876 0.793
0.982 0.772
0.789 0.737
0.995 0.720
0.828 0.677
0.595 0.586
0.677 0.468
AUC type:
Internal
External
AUC:
0.5
0.6
0.7
0.8
0.9
B
Test Set
Average
Training Set
PRJEB10878
PRJEB27928
PRJEB7774
PRJEB12449
PRJDB4176
PRJEB6070
PRJNA447983
PRJEB10878 0.80 0.88 0.78 0.66 0.82 0.76 0.77 0.78
PRJEB27928 0.82 0.85 0.78 0.68 0.72 0.75 0.80 0.76
PRJEB7774 0.73 0.79 0.86 0.63 0.74 0.71 0.70 0.72
PRJEB12449 0.75 0.72 0.82 0.60 0.76 0.77 0.71 0.76
PRJDB4176 0.83 0.80 0.72 0.66 0.70 0.72 0.70 0.74
PRJEB6070 0.72 0.75 0.76 0.59 0.58 0.84 0.54 0.66
PRJNA447983 0.77 0.87 0.81 0.68 0.79 0.69 0.80 0.77
AUC
1.0
0.9
0.8
0.7
0.6
0.5
0.4

Supplementary Figure 3. **Detailed comparison of internal- and external AUCs of different intestinal diseases.**

A, Comparation of internal and external AUCs with intra-cohort modeling between different intestinal diseases. The colored horizontal lines represent different AUC levels. The numbers marked in the bottom represent the mean of the corresponding AUCs. **B,** Heatmap showing cross validation AUCs on CRC datasets with intra-cohort modeling. Darker color indicates high AUC and bright color indicates low AUC. Gray indicates AUC $\leq$ 0.4. Diagonal values (i, i) represent 3 times repeated fivefold stratified internal cross-validations under project i. Non-diagonal values (i, j) represent external validations where project i as the training dataset and project j as the testing dataset. Right boxes indicate the average of each row values excluded diagonal value.

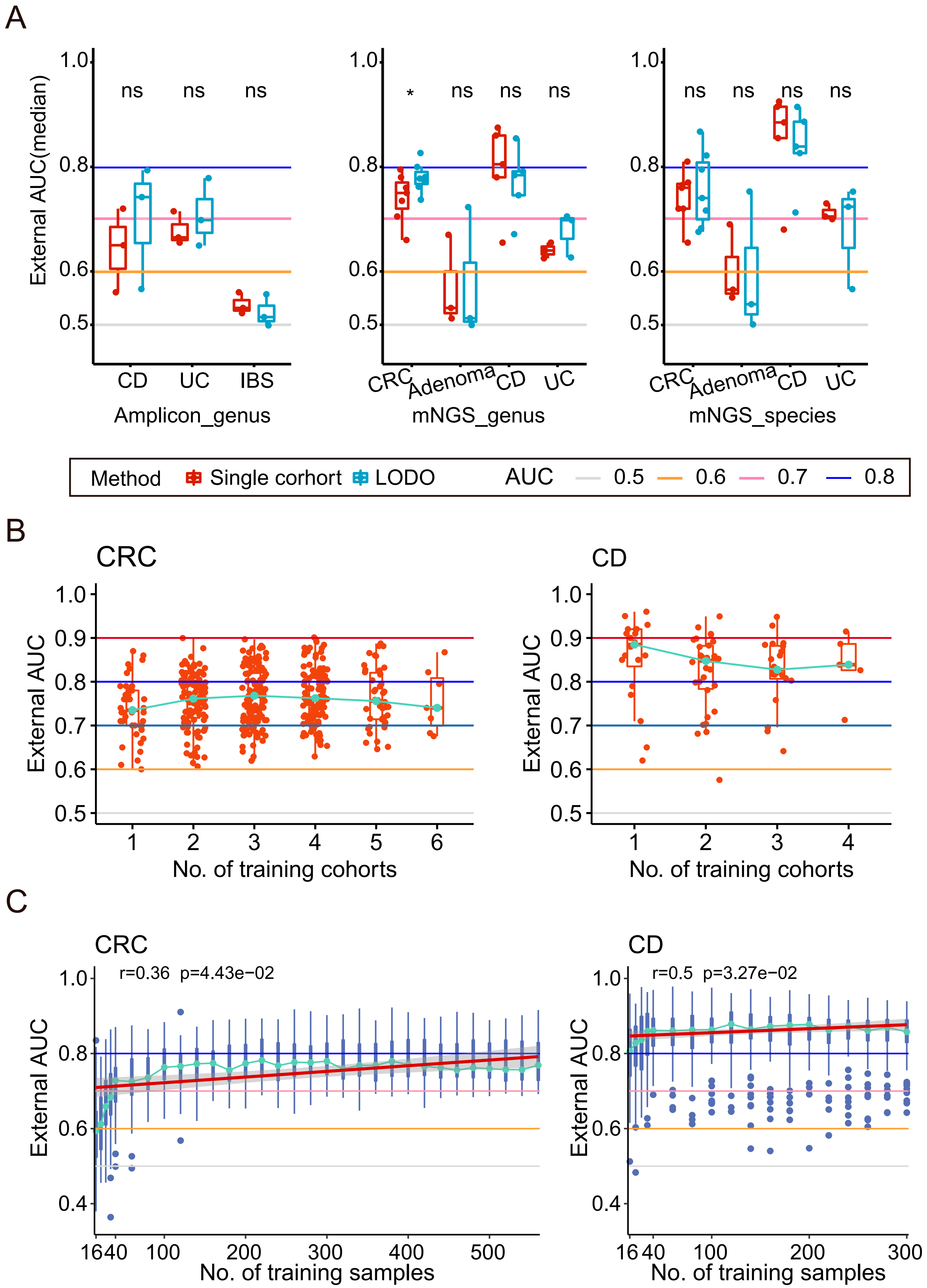
A
External AUC(median)
1.0
0.8
0.6
0.5
ns
ns
ns
*
CD
UC
IBS
Amplicon_genus
CRC
Adenoma
CD
UC
mNGS_genus
mNGS_species
Method
Single corhort
LODO
AUC
0.5
0.6
0.7
0.8
B
CRC
CD
External AUC
0.9
0.7
No. of training cohorts
C
r=0.36 p=4.43e−02
r=0.5 p=3.27e−02
0.4
No. of training samples
16
40
100
200
300
400
500

Supplementary Figure 4. **Strategies to improve external validation performance using LODO and Cohort-Cumulation modeling in intestinal diseases.**

A, Comparation of median external validation AUCs between intra-cohort and LODO modeling method per intestinal disease. Each point represents the median external AUC of each cohort (as testing dataset). Two-side paired Wilcoxon rank sum test was used for pairwise group comparisons. **B,** External AUCs for the testing datasets at increasing numbers of training cohorts considered for the model (CCM). Intestinal diseases with more than or equal to 5 were shown here (including CRC and CD). The green line linked the median external AUC at each number of training datasets. **C,** External AUCs for the LODO modeling at increasing numbers of samples considered for the training model (SCM). Intestinal diseases with more than or equal to 5 were shown here (including CRC and CD). The green line linked the median external AUC at each number of training datasets. The red line represents the linear regression model of the No. of training samples to median external AUC (Table S4), and Spearman correlation analysis was also carried out (correlation coefficient and p value were shown at the top).

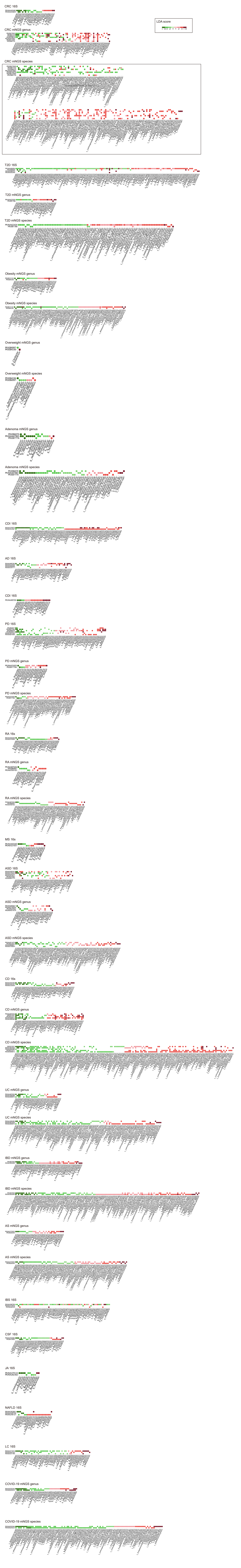
CRC 16S
CRC mNGS genus
CRC mNGS species
LDA score
T2D 16S
T2D mNGS genus
T2D mNGS species
Obesity mNGS genus
Obesity mNGS species
Overweight mNGS genus
Overweight mNGS species
Adenoma mNGS genus
Adenoma mNGS species
CDI 16S
AD 16S
CDI 16S
PD 16S
PD mNGS genus
PD mNGS species
RA 16s
RA mNGS genus
RA mNGS species
MS 16s
ASD 16S
ASD mNGS genus
ASD mNGS species
CD 16s
CD mNGS genus
CD mNGS species
UC mNGS genus
UC mNGS species
IBD mNGS genus
IBD mNGS species
AS mNGS genus
AS mNGS species
IBS 16S
CSF 16S
JA 16S
NAFLD 16S
LC 16S
COVID-19 mNGS genus
COVID-19 mNGS species

Supplementary Figure 5. **The markers of 20 diseases**.

Markers were calculated by LEfSe analysis with an LDA cutoff of 2. The darker and lighter colors represent the larger and smaller absolute values of LDA respectively. Red indicates that marker is enriched in the disease group, while green indicates that marker is enriched in the healthy group.

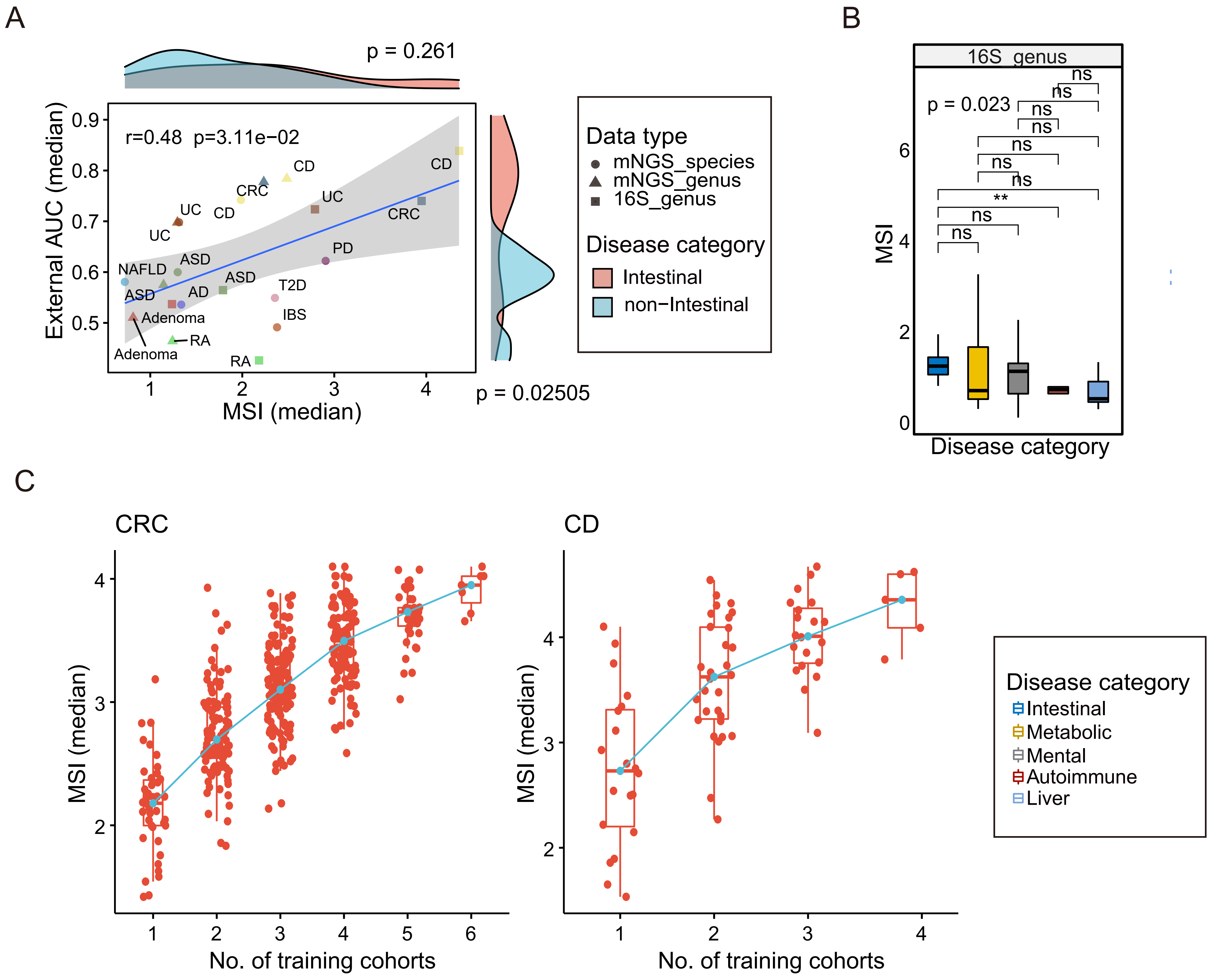
A
p = 0.261
r=0.48 p=3.11e−02
External AUC (median)
MSI (median)
p = 0.02505
Data type
mNGS_species
mNGS_genus
16S_genus
Disease category
Intestinal
non−Intestinal
CD
CRC
UC
PD
NAFLD
ASD
AD
T2D
IBS
Adenoma
RA
B
16S_genus
p = 0.023
ns
**
MSI
Disease category
C
CRC
CD
MSI (median)
No. of training cohorts
Disease category
Intestinal
Metabolic
Mental
Autoimmune
Liver

Supplementary Figure 6. **Association between the median of MSIs and external validation AUCs.**

A, Correlation between the median MSIs and external validation AUCs using LODO modeling of each disease (Spearman r=0.48 p=3.11e−02); The shape and color represent different data types and diseases. The x-axis value of each point represents the median of MSI when dataset under organization of LODO modeling in each disease, and the y-axis value represents the median external validation AUC using intra-cohort modeling in each disease. The density distributions of x- and y-axis between intestinal and non-intestinal diseases were shown at the top and right. Two-side paired Wilcoxon rank sum test was used for pairwise group comparisons. **B,** Boxplots of MSIs between different data types in each disease category. Dataset excluded IBS disease. The colors represent the different disease categories. Kruskal-Wallis test was used for multiple-group comparisons and p value was shown at the top of the picture. Multiple pairwise Wilcoxon rank sum test comparisons are adjusted and p values were shown above the line segment. Box elements show the median and upper and lower quartiles. **C,** Median MSIs calculated from dataset under organization of CCM. The green line linked the median MSI at each number of training datasets. Intestinal diseases with more than or equal to 5 were shown here (including CRC and CD).

*$p < 0.05$, **$p < 0.01$, ***$p < 0.001$, ****$p < 0.0001$

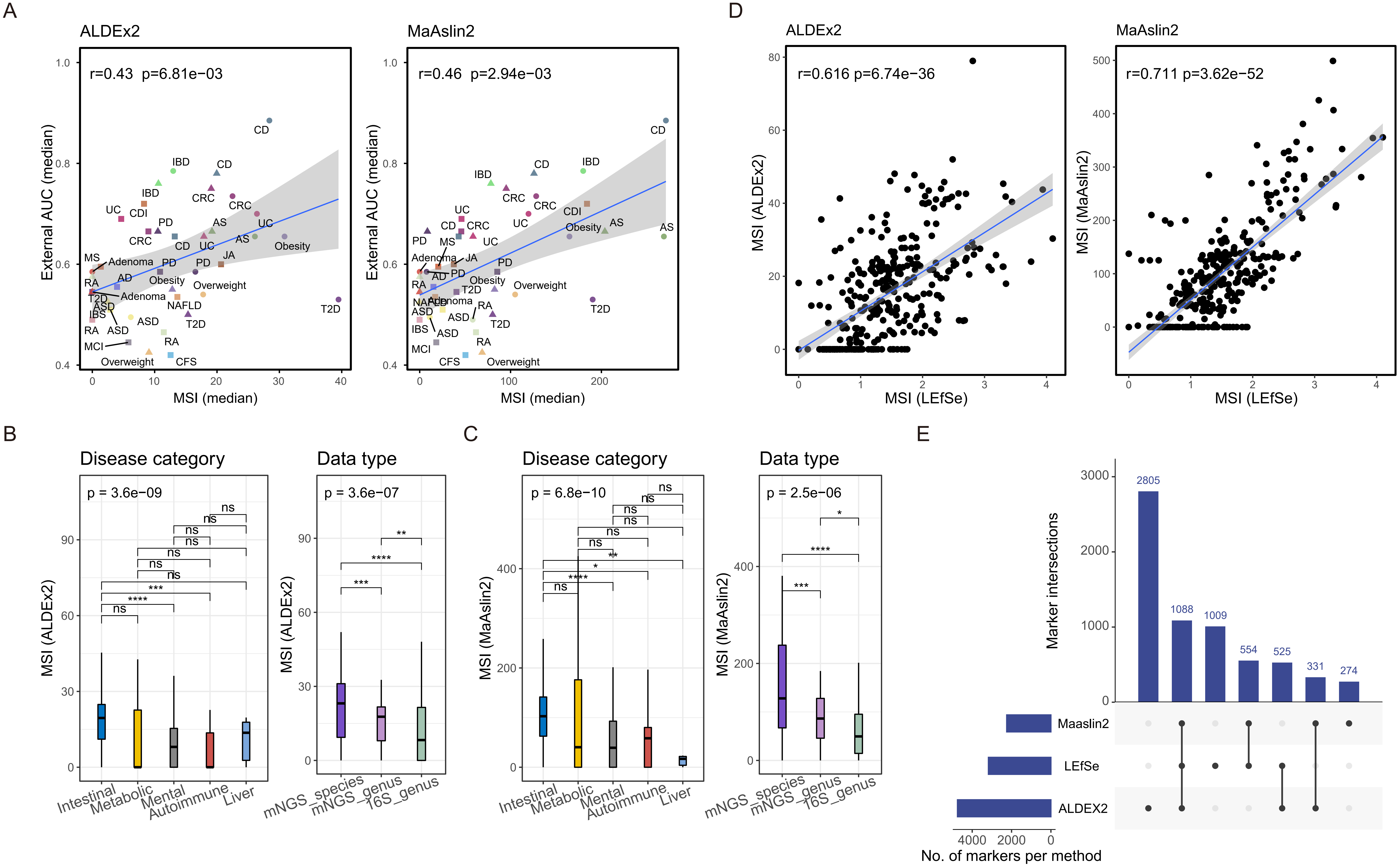
A
ALDEx2
r=0.43 p=6.81e−03
External AUC (median)
MSI (median)
MaAslin2
r=0.46 p=2.94e−03
External AUC (median)
MSI (median)
D
ALDEx2
r=0.616 p=6.74e−36
MSI (ALDEx2)
MSI (LEfSe)
MaAslin2
r=0.711 p=3.62e−52
MSI (MaAslin2)
MSI (LEfSe)
B
Disease category
p = 3.6e−09
MSI (ALDEx2)
Intestinal
Metabolic
Mental
Autoimmune
Liver
Data type
p = 3.6e−07
MSI (ALDEx2)
mNGS_species
mNGS_genus
16S_genus
C
Disease category
p = 6.8e−10
MSI (MaAslin2)
Intestinal
Metabolic
Mental
Autoimmune
Liver
Data type
p = 2.5e−06
MSI (MaAslin2)
mNGS_species
mNGS_genus
16S_genus
E
Marker intersections
2805
1088
1009
554
525
331
274
Maaslin2
LEfSe
ALDEX2
No. of markers per method

Supplementary Figure 7. **Comparation of MSIs calculated by three differential abundance analysis methods.**

A, Left: Correlation between the ALDEx2-based median MSIs (x-axis) and external validation AUCs (y-axis; Spearman $r = 0.43$ $p = 6.81e{-}03$); The shape and color represent different data types and diseases. Right: Correlation between the MaAsLin2-based median MSIs and external validation AUCs (Spearman $r = 0.46$ $p = 2.94e{-}03$). **B,** Left: Comparation of ALDEx2-based MSIs between different disease categories. Right: Comparation of ALDEx2-based MSIs between different data types, where the diseases that did not have all three data types were excluded. Kruskal-Wallis test was used for multiple-group comparisons and the *p* value was shown at the top of the plot. Multiple pairwise Wilcoxon rank sum test comparisons are adjusted and *p* values were shown above the line segment. Box elements show the median and upper and lower quartiles. **C,** Left: Comparation of the MaAsLin2-based MSIs between different disease categories. Right: Comparation of the MaAsLin2-based MSIs between different data types, where the diseases that did not have all three data types were excluded. **D,** Left: Correlation between the LEfSe- and ALDEx2- based MSIs (Spearman $r = 0.616$ $p = 6.74e{-}36$); Right: Correlation between the LEfSe- and MaAsLin2 - based MSIs (Spearman $r = 0.711$ $p = 3.62e{-}52$); **E,** Overlaps in markers calculated by ALDEx2, LEfSe and MaAsLin2.

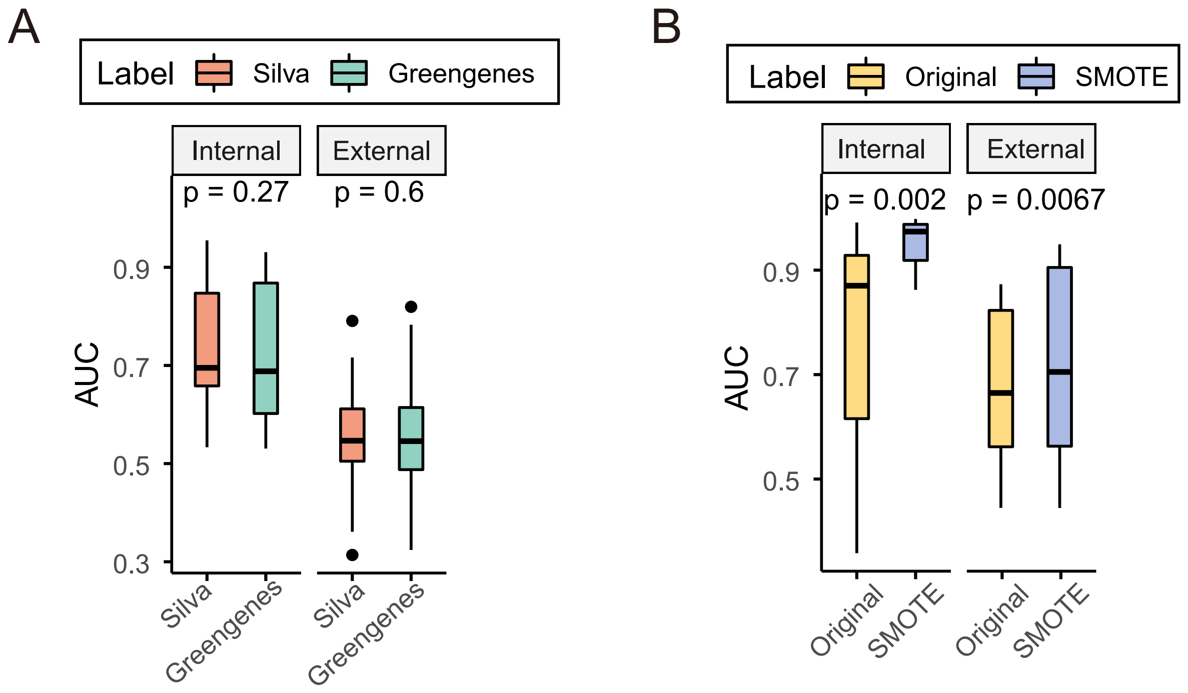
A
Label
Silva
Greengenes
Internal
External
p = 0.27
p = 0.6
AUC
0.9
0.7
0.5
0.3
Silva
Greengenes
Silva
Greengenes
B
Label
Original
SMOTE
Internal
External
p = 0.002
p = 0.0067
AUC
0.9
0.7
0.5
Original
SMOTE
Original
SMOTE

Supplementary Figure 8. **Comparation of AUCs modeled by microbiome data classified against the Silva and Greengene database, and processed by SMOTE before and after.**

A, Comparation of AUCs (internal and external) calculated by the microbiome data classified against the Greengenes and Silva databases. Two-side paired Wilcoxon rank sum test was used for pairwise group comparisons. **B,** Comparation of AUCs (internal and external) calculated by the microbiome data processed by SMOTE before and after.

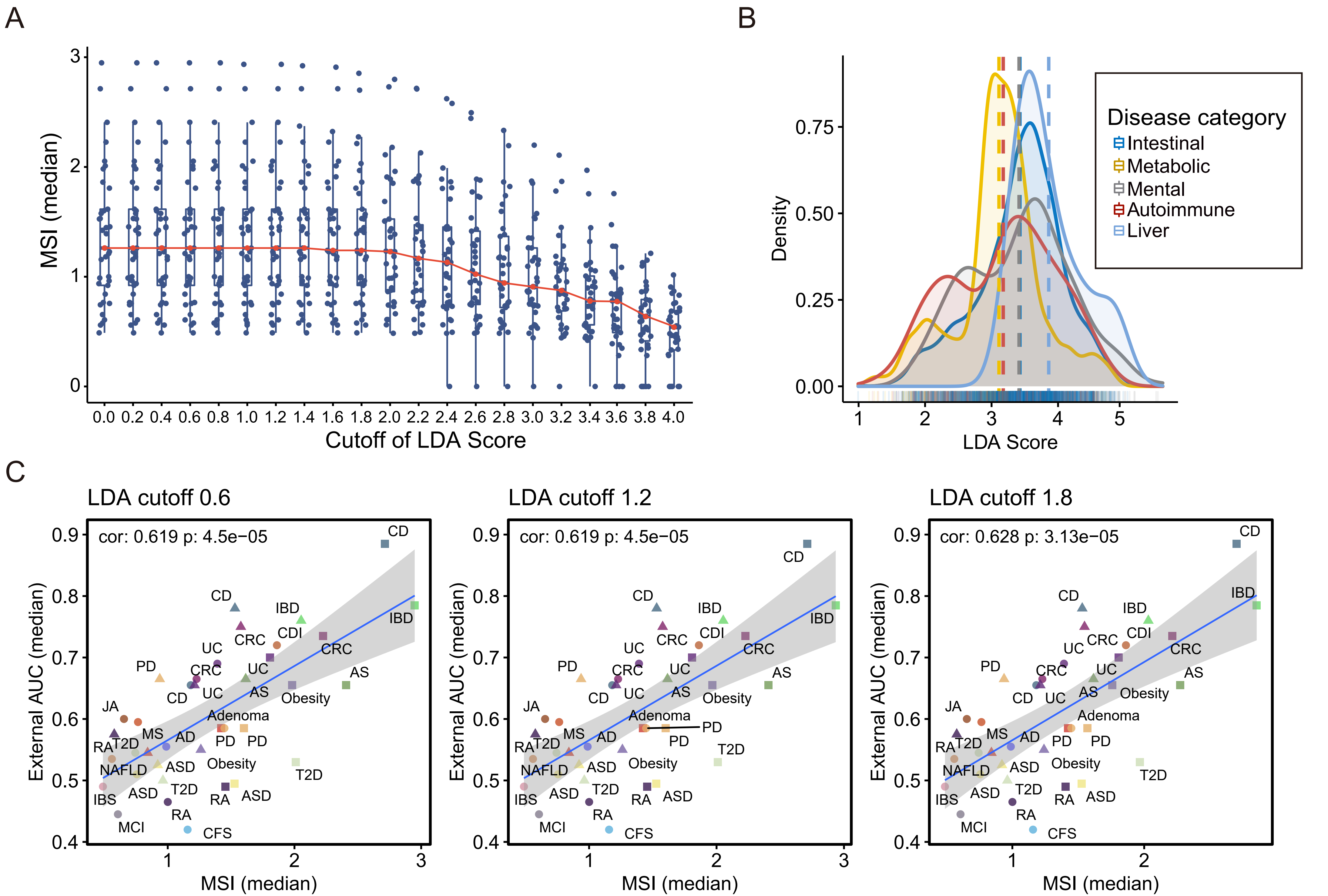

A
MSI (median)
Cutoff of LDA Score
B
Density
LDA Score
Disease category
Intestinal
Metabolic
Mental
Autoimmune
Liver
C
LDA cutoff 0.6
cor: 0.619 p: 4.5e−05
LDA cutoff 1.2
cor: 0.619 p: 4.5e−05
LDA cutoff 1.8
cor: 0.628 p: 3.13e−05
External AUC (median)
MSI (median)

Supplementary Figure 9. **Influence of different LDA cutoffs on MSI and its correlation with external AUCs.**

A, Boxplots of MSIs with different LDA cutoffs from 0 to 4. Datasets from the Overweight were excluded for only two markers were obtained at the genus level. The red line links the median MSI at each LDA cutoff. Box elements show the median and upper and lower quartiles. **B,** Density plot of the LDA scores among different disease categories. **C,** Correlation between the median MSIs under different LDA cutoffs (including 0.6, 1.2 and 1.8) and external validation AUCs using intra-cohort modeling of each disease; The shape and color represent different data types and diseases. The x-axis value of each point represents the median MSI values for each dataset, and the y-axis value represents the median external validation AUCs of the intra-cohort classifier on other datasets of the same disease. Spearman correlation analysis was also carried out between the MSI and external AUC values (the correlation coefficients and *p* values were shown at the top).

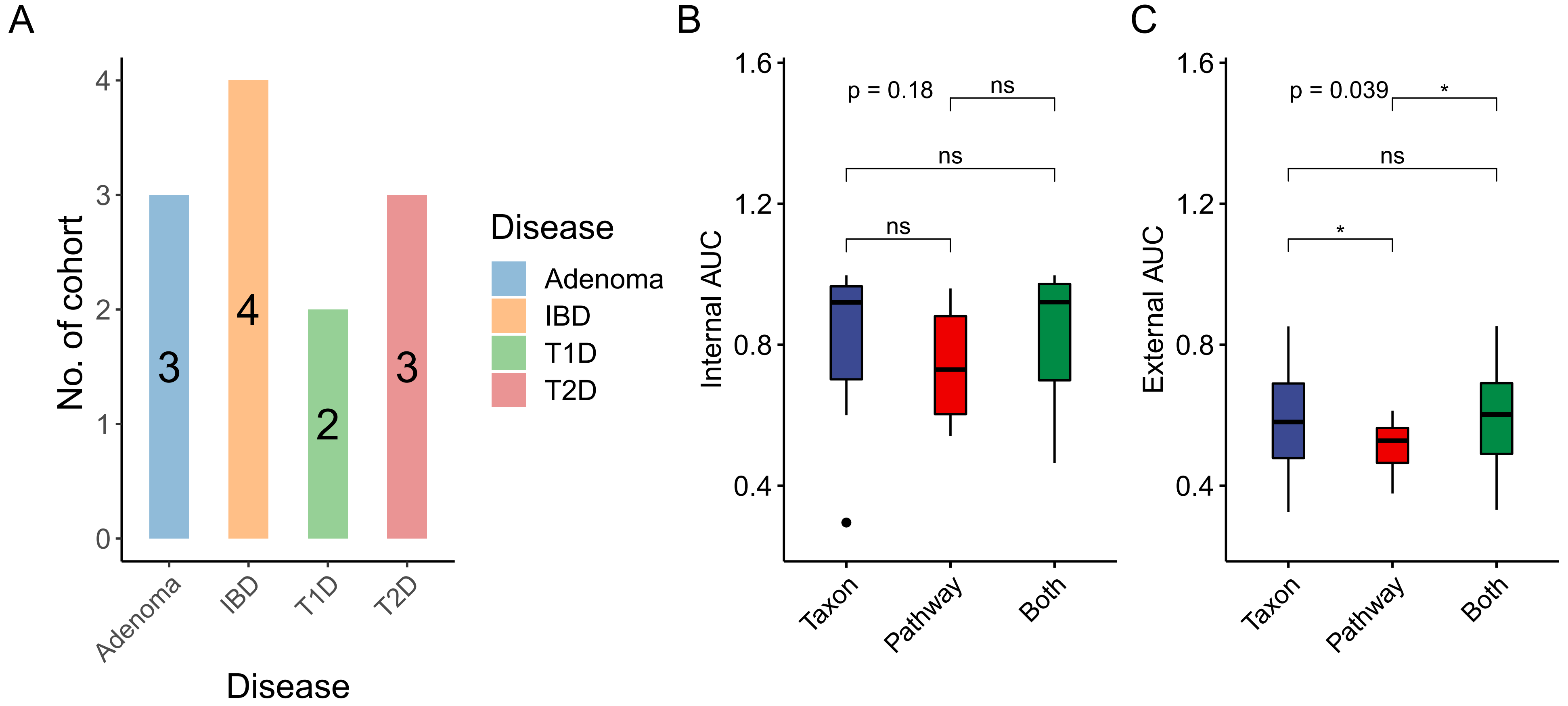
A
B
C
4
3
2
1
0
3
4
2
3
No. of cohort
Adenoma
IBD
T1D
T2D
Disease
Disease
Adenoma
IBD
T1D
T2D
1.6
1.2
0.8
0.4
Internal AUC
p = 0.18
ns
ns
ns
Taxon
Pathway
Both
External AUC
p = 0.039
*
ns
*

Supplementary Figure 10. **Comparation of classifier performance using taxonomic and/or function data from the ‘curatedMetagenomicData’ R package.**

A, Diseases and corresponding cohorts obtained from the ‘curatedMetagenomicData’ R package. Here four diseases which have at least 3 cohorts in the ‘curatedMetagenomicData’ were retrained. **B,** Comparation of the internal AUCs with intra-cohort modeling between using taxon, pathway abundances and both combined. **C,** Comparation of the external AUCs with intra-cohort modeling between using taxon, pathway abundances and both combined. **D,** Comparation of the internal AUCs with intra-cohort modeling between using taxon, pathway and combined abundance data. **E,** Comparation of the external AUCs with intra-cohort modeling between using taxon, pathway and combined abundance data. Kruskal-Wallis test was used for multiple-group comparisons. Multiple adjusted two sides Wilcoxon rank sum test was used for pairwise group comparisons.
