# Supplementary material for: Performance of Gut Microbiome as an Independent Diagnostic Tool for 20 Diseases: Cross-Cohort Validation of Machine-Learning Classifiers
Source: Gut Microbes. 2023 May 4;15(1):2205386. doi: 10.1080/19490976.2023.2205386 (PMC10161951; doi:10.1080/19490976.2023.2205386)
Supplement: Supplemental Material [file KGMI_A_2205386_SM1540.zip › Supplementary Material/Supplementary Tables description.docx]

**Supplementary Tables**

1. Table S1. Overview of diseases, datasets and meta information after filtering.
2. Table S2. The internal and external validations with intra-cohort modeling of all disease.
3. Table S3. Different two-factor (disease category and data type) ANOVA format of the external validation AUCs (Unbalanced design) with intra-cohort modeling.
4. Table S4. The linear regression models for the number of training sample to external AUCs (median) by SCM.
5. Table S5. Comparation between our intra-cohort validation AUCs and corresponding literatures’ and re-evaluation of the excellent validation results of other literatures.
6. Table S6. The internal and external validations AUC-PR and MCC with intra-cohort modeling of all disease.
7. Table S7. The internal and external cross validations with intra-cohort modeling with both taxon, pathway and both combined relative abundance from “curatedMetagenomicData” R package.
